# Aspirin-Based Organoiron Dendrimers as Promising Anti-Inflammatory, Anticancer, and Antimicrobial Drugs

**DOI:** 10.3390/biom11111568

**Published:** 2021-10-22

**Authors:** Alaa S. Abd-El-Aziz, Maysun R. Benaaisha, Amani A. Abdelghani, Rabin Bissessur, Laila H. Abdel-Rahman, Ahmed M. Fayez, Doaa Abou El-ezz

**Affiliations:** 1 Department of Chemistry, University of Prince Edward Island, 550 University Avenue, Charlottetown, PE C1A 4P3, Canada; mbenaaisha@upei.ca (M.R.B.); aabdelghani@upei.ca (A.A.A.); rabissessur@upei.ca (R.B.); 2 Chemistry Department, Faculty of Science, Sohag University, Sohag 82524, Egypt; Laila.abdelrahman@science.sohag.edu.eg; 3 School of Life and Medical Sciences, University of Hertfordshire Hosted by Global Academic Foundation, New Administrative Capital, Cairo 11835, Egypt; afayez@herts.ac.uk; 4 Department of Pharmacology and Toxicology, Faculty of Pharmacy, October University for Modern Sciences and Arts (MSA University), Giza 8655, Egypt; Dabulez@msa.edu.eg

**Keywords:** dendrimer, aspirin, in vivo and in vitro anti-inflammatory activity, anticancer activity, gastrointestinal toxicity

## Abstract

Designing nanocarriers with actions directed at a specific organ or tissue is a very promising strategy since it can significantly reduce the toxicity of a bioactive drug. In this study, an organometallic dendrimer was used to synthesize a biocompatible drug delivery system by attaching aspirin to the periphery of the dendrimer. Our goal is to enhance the bioavailability and anticancer activity of aspirin and reduce its toxicity through successive generations of organoiron dendrimers. The biological activity of aspirin-based dendrimer complexes was evaluated. The result of antimicrobial activity of the synthesized dendrimers also demonstrated an increase in their antimicrobial activity with increased generation of the dendrimers for most types of microorganisms. This study reveals for the first time that organoiron dendrimers linked with aspirin exhibit an excellent Gram-negative activity comparable to the reference drug Gentamicin. All synthesized dendrimers were tested for their anticancer activity against breast cancer cell lines (MCF-7), hepatocellular cell lines (Hep-G2), and a non-cancer cell line, Human Embryonic Kidney (HEK293), using the MTT cell viability assay and compared against a standard anticancer drug, Doxorubicin. Compounds **G3-D9-Asp** and **G4-D12-Asp** exhibited noticeable activity against both cell lines, both of which were more effective than aspirin itself. In addition, the in vivo anti-inflammatory activity and histopathology of swollen paws showed that the designed aspirin-based dendrimers displayed significant anti-inflammatory activity; however, **G2-D6-Asp** showed the best anti-inflammatory activity, which was more potent than the reference drug aspirin during the same period. Moreover, the coupling of aspirin to the periphery of organoiron dendrimers showed a significant reduction in the toxicity of aspirin on the stomach.

## 1. Introduction

Dendrimers constitute an interesting class of macromolecules that find applications in various fields, such as catalysis, electronics, and biomedicine [1,2]. Indeed, dendrimers could pave the way for novel therapeutic approaches [3]. Dendrimers are hyperbranched molecules whose size, topology, and flexibility can be strictly controlled during their synthesis.4 This allows for various functional groups to be grafted onto the outer shell of dendrimers, which can then interact with other (macro) molecules [4,5]. This functionalization can be easily tuned to develop biocompatible and versatile products [6,7,8,9]. In addition, their unique properties, including globular shape, nanoscale size, internal cavities, high reactivity, and convenient synthesis procedures, make dendrimers promising agents in pharmaceutical fields [10,11]. Drugs covalently bonded to the periphery of dendrimers enhance drug stability and provide better control of their release, which can reduce drug toxicity while increasing its efficacy [12,13]. Dendrimer-based drug delivery systems have emerged to be the most promising systems for meeting the needs of an ideal drug delivery system. Dendrimers have been shown to be a suitable candidate for oral drug administration, as they loosen tight junctions in epithelial cells, allowing for better absorption of small molecular weight drugs [7,14]. Early dendrimer-based drug delivery systems were mainly organic and derived their activities from conjugated drug molecules [15,16]. Although organic dendrimers continue to attract attention, metal-based dendrimers are being explored as an alternative [17,18,19]. The combination of transition metal ions with dendrimers provides highly ordered macromolecular structures with engaging, magnetic, electronic, photo-optical, and bioactivity properties [15,16,17,18,19,20,21,22]. Metal-based macromolecules have been proven to be effective against various microorganisms. Toward this, our research group has focused on incorporating *η6*-aryl-*η5*-cyclopentadienyliron (II) complexes into dendrimers structure, which has been extensively used for various applications, especially in the biomedicine field [6,23,24]. The use of the organoiron sandwich complexes to construct several dendrimers has led to the development of novel drug carriers with potent activity against infection-causing microorganisms. Indeed, the presence of redox-active iron centers in the dendrimers structure has a key role in activating the formation of reactive oxygen species (ROS), which in turn eliminate microorganisms [25,26].

Aspirin is a non-steroidal anti-inflammatory drug (NSAID) widely used worldwide [27]. More than 82 million aspirin tablets are consumed each year [28]. Aspirin (acetylsalicylic acid) is a white crystalline acidic product with great analgesic, antipyretic, and anti-inflammatory activities [29,30]. The demand for aspirin and its derivatives for other biological uses is increasing due to their availability and their ability to act as a precursor to further modification via the carboxyl group [31]. Aspirin’s therapeutic mechanism has been attributed to the irreversible inhibition of cyclooxygenase (COX), which is a key enzyme for catalyzing the formation of prostaglandins (PG), including PGE2, PGD2, and thromboxane A2 (TXA2) [32,33,34]. These agents are known to trigger inflammation, pain, fever, and blood clotting [32,34]. Aspirin has attracted the attention of several researchers in terms of its role in preventing cancer cell growth in recent decades. Several studies have shown that aspirin exhibits protective effects mainly against gastric, esophageal, prostate, and hepatocytic cancers [35,36,37,38]. Many clinical studies also indicated that taking aspirin may reduce the risk of in situ breast cancer [38]. However, long-term consumption of aspirin is associated with severe side effects such as gastrointestinal bleeding and hemorrhagic stroke [36,38]. Over the past two decades, many efforts have been made to develop selective aspirin derivatives that can reduce these side effects. For instance, the chemical modification of aspirin has enhanced its pharmacological properties with reduced toxicity in the gastrointestinal tract [39,40]. Aspirin derivatives also showed antibacterial activities against *Pseudomonas aeruginosa, Escherichia coli, Bacillus subtilis,* and *Staphylococcus aureus* [41,42]. Other pharmacological activities of aspirin derivatives were also reported, such as anticoagulant, anti-fungal, antiplatelet, and anticancer abilities [43,44,45]. The pharmacological properties of aspirin and other drugs can also be enhanced by forming biodegradable polymer-drug conjugates. Many polymer-drug conjugates with non-steroidal anti-inflammatory (NSAID) drugs have been reported using a wide range of polymers, such as poly(anhydride-esters), dendrimers, dextran, copolymers of 2-hydroxyethyl methacrylate (HEMA), and oligo(3-hydroxybutanoate) [46,47,48,49,50]. Coupling of NSAIDs with the polymeric carrier is aimed at obtaining new forms of drugs with beneficial properties such as increased cellular uptake rate, optimized drug biodistribution, increased stability, prolonged drug release, increased solubility, and decreased toxicity [9,51,52,53]. In the present study, we thereby aimed to enhance the therapeutic efficacy of aspirin and decrease its toxicity through successive generations of organometallic dendrimers capped with a high number of terminal aspirin molecules. Dendritic structures were analyzed by spectroscopic and elemental analysis, which were also used to distinguish between dendrimer generations as well as the terminal groups of the same generation. The morphology of the dried residues of the dendrimers was also determined using scanning electron microscopy (SEM). In addition, the thermal stability, electrochemical behaviors, and biological activities associated with these complexes have been characterized.

## 2. Methods and Materials

All used chemicals, including aspirin, were obtained from Sigma-Aldrich. The chemicals, unless otherwise indicated, were used without any further purification. The solvents were dried and stored over 3Å molecular sieves before being utilized. The five NSAID drugs were prepared at the Department of Chemistry, University of Prince Edward Island. All other chemicals used were analytically graded. The design of the organoiron complex **1** was carried out by following previously reported procedures [54,55,56]. Four generations of dendrimer were synthesized based on previously reported procedures [6,57].

### 2.1. Instrumentation

A Bruker Avance NMR spectrometer (^1^H, 400 MHz and ^13^C, 100 MHz) was utilized to characterize all synthesized dendrimers in DMSO-d_6_ with the chemical signals referenced to solvent residual signal in ppm. Fourier transform absorption spectroscopy (ATR-FTIR) measurements were obtained on a Brooker Alpha-P FTIR spectrophotometer. Cyclic voltammetric experiments were performed on a Princeton Applied Research/EG&G Model 263 potentiostat/galvanostat utilizing glassy carbon working electrode, Pt counter electrode, and Ag reference electrode. Experiments were achieved at a scan rate of 0.1 to 1.5 V/s and at 25 °C and −25 °C in an atmosphere of nitrogen in degassed propylene carbonate as a solvent and tetrabutylammonium hexafluorophosphate as supporting electrolyte and were extremally referenced to the DMF solution of ferrocene. Scanning electron micrographs (SEM) of the prepared dendrimers were acquired on Hitachi TM3000 SEM. Elemental analyses were also performed on CE-440 Elemental Analyser, Exeter Analytical, Inc.

### 2.2. Biological Measurements

#### 2.2.1. Antimicrobial Assay

The agar well diffusion method was used in the measurement of the antimicrobial activity of synthesized dendrimers. All the dendrimers were tested in vitro for their antibacterial activity against Gram-positive bacteria, *Bacillus subtilis* (ATCC 6051) and *Staphylococcus aureus* (ATCC12600)*,* Gram-negative bacteria *Escherichia coli* (ATCC 1175) and *Klebsiella pneumoniae* (ATCC10145). Ampicillin and gentamicin were utilized as standard drugs against Gram-positive and Gram-negative bacteria, respectively. For the two yeasts, *Candida albicans* and *Aspergillus niger*, nystatin was used as a standard drug. DMSO was utilized as solvent control. The prepared complexes were assessed at a concentration of 15 mg/mL against both bacterial and fungal strains.

#### 2.2.2. Method of Testing

The sterile medium was poured onto the sterile Petri dishes (20–25 mL, each Petri dish) and left to harden at room temperature. The microbial suspension was prepared in sterile saline equivalent to McFarland 0.5 (1.5 × 105 CFU mL^−1^) standard solution, and its turbidity was modified to OD = 0.13 applying spectrophotometer at 625 nm. Optimally, within 15 min after modifying the turbidity of the inoculum suspension, a sterilized cotton swab was dipped into the modified suspension and was occupied on the dried agar surface, then left to dry for 15 min with cover in place. Wells of 6 mm in diameter were produced in the solidified media with the support of a sterilized borer. A total of 100 μL of the solution of the tested compound was added to each well with the help of a micropipette. The plates were stored at 37 °C for 24 h before assessment. This assessment was performed in triplicate, and areas of inhibition were measured on the mm scale.

#### 2.2.3. MTT Assay

The cytotoxic activities of the tested dendrimers against the breast cancer cell line MCF-7, embryonic kidney non-cancer cells (HEK-293), and hepatic cellular Hep-G2 were evaluated by MTT assay. A cell suspension was diluted with complete medium to a concentration of 5 × 104 cell/ml. By using a micropipette, 100 μL aliquots of the cell suspension were pipetted into all wells of 96-well plate (~5000 cells/well). These plates were incubated at 37 °C for one day to allow cell attachment. Then, cells were treated using 100 µL of growth medium consisting of 0, 0.001, 0.01, 0.1, 1, 10, or 100 μL of the freshly prepared aspirin-based dendrimers in triplicate. After that, cells were rinsed with phosphate-buffered saline (PBS), and an appropriate fresh medium including 20 μL MTT in phosphate-buffered saline (0.5 mg/mL) was added to the test wells. The 96-well plate was then further incubated in a carbon dioxide incubator at 37 °C for another 4 h, and the MTT assay study was done. The MTT assay procedure is based on the reduction of the tetrazolium salt MTT to unsolvable purple formazan via metabolically effective cells, making their activities quantifiable via spectrophotometry. Consequently, the produced formazan crystals were dissolved in 120 μL DMSO solvent for each well. Cell viability was established by measuring the absorbance of each well at 570 nm, and at a reference wavelength of 630 nm using an ELISA plate reader. Findings are expressed in terms of the concentration required to inhibit cell growth by 50% relative to untreated cells (*IC*_50_). Vinblastine was used as the standard and the corresponding *IC*_50_ values were determined by Equation (1):

(1) $$IC_{50}\left(\%\right)=\frac{Control_{OD}-Compound_{OD}}{Control_{OD}}x\;100$$

#### 2.2.4. Animals

The present study was performed on adult albino Wistar rats, weighing 185 ± 200 g. Animals were kept in the animal house at MSA university. They were kept under suitable conditions of humidity and temperature (humidity 60–70%, temperature 24 ± 2 °C). The animals were fed by standard pellet chow (El-Nasr chemical Co., Cairo, Egypt) and were given access to water *ad libitum*. 

### 2.3. Anti-Inflammatory Activity

#### 2.3.1. In Vitro COX-1 and COX-2 Inhibition Assay

All generations of the new synthesized drug functionalized dendrimers were screened for their in vitro cyclooxygenase (COX-1 & COX-2) inhibitory activity compared to the positive drugs aspirin, diclofenac sodium, indomethacin, rofecoxib, and celecoxib using an ovine COX-1/COX-2 assay kit. The selectivity index (SI values) was defined as IC_50_ COX-1/ IC_50_ COX-2.

#### 2.3.2. Assessment of In Vivo Anti-Inflammatory Activity

##### In Vivo Rat Paw Edema Assay

Inflammation was induced in all groups by carrageenan (0.2 mL, 1% saline solution), which was injected subcutaneously into the plantar surface of the right hind paw. Group 1 served as a normal group, group 2 received carrageenan only, and groups 3, 4, 5, 6, and 7 received the test drugs orally 1 h before carrageenan (*n* = 6) [58]. Edema was measured in diameter (mm) at 1 h, 2 h, 4 h, and 6 h after carrageenan injection using a digital caliper. Percentage edema inhibition was calculated as [1 − (sample diameter/control diameter)] × 100. All data of the paw edema are represented as mean ± SD. Statistical significance was measured at *p* < 0.05 based on one-way analysis of variance ANOVA test followed by Tukey’s test for multiple comparisons. **b** was significantly different from the control group at *p* < 0.05.

##### Determination of Rat Serum PGE2

Inflammation was induced in all groups by carrageenan (0.2 mL, 1% saline solution) injected subcutaneously into the plantar surface of the right hind paw. Group 1 served as a normal group, group 2 received carrageenan only, and groups 3, 4, 5, 6, and 7 received the test drugs orally 1 h before carrageenan (*n* = 6) [58]. Blood samples were collected from all groups and centrifuged for 15 minutes to separate the serum and measure serum PGE2 Pg/mL. Percentage inhibition was calculated as [1 − (sample PGE2 concentration/control PGE2 concentration)] × 100. All data of the PGE2 concentration are represented as mean ± SD. Statistical significance was measured at *p* < 0.05 based on one-way analysis of variance ANOVA test followed by Tukey’s test for multiple comparisons. **b** was significantly different from the control group at *p* < 0.05.

##### Evaluation of Gastrointestinal Toxicity of the Tested Dendrimers

The gastric mucosa was carefully inspected for the occurrence of ulcers with the aid of an illuminated magnifying lens (l0×), the ulcer score was calculated according to published method [59] and ulcer index was determined using the reported formula [60]. Ulcer index = 10/x, where x= total mucosal area/ total ulcerated area. All data are represented as mean ± standard deviation. Statistical significance was considered at *p* < 0.05 based on one-way analysis of variance ANOVA test followed by Tukey’s test for multiple comparisons. **a** significantly different from normal at *p* < 0.05. **b** was significantly different from the aspirin group at *p* < 0.05.

##### Experimental Design

Rats were fasted for 24 h and then randomly allocated into the following groups to evaluate the potential toxicity of dendrimers on the stomach:

Group 1 served as normal group. 

Group 2 received aspirin (400 mg/kg) orally.

Group 3 received G1 (400 mg/kg) orally.

Group 4 received G2 (400 mg/kg) orally.

Group 5 received G3 (400 mg/kg) orally.

Group 6 received G4 (400 mg/kg) orally.

Animals were left for 4 h and then sacrificed under light anesthesia for stomach dissection. The gastric mucosa was carefully inspected for the occurrence of ulcers with the aid of an illuminated magnifying lens (l0x), the ulcer score was calculated based on published study [59] and ulcer index was determined using the reported formula [60]. Ulcer index = 10/x, where x= total mucosal area/total ulcerated area.

##### Histopathology Examination

The stomach was dissected out, rinsed, and kept in a 10% neutral formalin solution. After fixation, tissues were routinely processed and stained with hematoxylin and eosin for light microscopy [61]. Lesion scores were evaluated in this study according to a published report [62] three parameters were evaluated in relation to their severity; epithelial cell loss was given a score from 0 to 3, hemorrhage was given a score from 0 to 4, and a score from 0 to 2 was given for inflammatory cell infiltration. The total score was given by the summation of the three scores.

##### Statistical Analysis

Comparison between means was made using one-way analysis of variance (ANOVA) followed by Tukey’s multiple comparisons test. For all statistical tests, the level of significance was fixed at *p* < 0.05. Statistical tests were carried out using GraphPad Prism software package, version 5a (GraphPad Software, Inc., San Diego, CA, USA).

## 3. Synthesis and Characterization

### 3.1. General Procedure

Four generations of Aspirin based-dendrimers (**G1-D3-Asp, G2-D6-Asp**, **G3-D9-Asp,** and **G4-D12-Asp)** were synthesized using Steglich esterification [56], using the proper molar ratio of the core, drugs, DMAP, and DCC. The solutions were stirred at 0 °C under a nitrogen atmosphere for 15 min. After that, the reaction mixture was warmed to room temperature and left to stir for two days. The mixture was cooled to −25 °C for 2 h, filtered to remove dicyclohexylurea (DHU), and after that poured into 10% HCl solution. The products were dried, then dissolved in acetone, cooled to −25 °C in a freezer for two h, filtered to remove the remaining DHU, and the removal of the solvent gave rise to the products. The methodology was used to synthesize dendrimer **G1-D3-Asp** using a 1:6 molar ratio, dendrimer **G2-D6-Asp** using a 1:12 molar ratio, dendrimer **G3-D9-Asp** using a 1:24 molar ratio, and finally, dendrimer **G4-D12-Asp** was synthesized using a 1:48 molar ratio. Detailed synthetic methodologies, yields %, and characterization using ^1^H and ^13^C NMR, ATR-FTIR, and elemental analyses are reported here. 

### 3.2. Aspirin-Terminated G1-D3-Asp Dendrimer

The Steglich esterification reaction was used to conjugate aspirin with the first generation of dendrimer. A 25 mL round-bottom flask was charged with **G1-D2** (0.25 g, 0.07 mmol), **Aspirin** (0.21 g, 1.21mmol), DMAP (0.28 g, 2.23 mmol), 10 mL of DMF, and after 10 min the DCC (0.24 g, 1.16 mmol) was added (molecular weight 4687.9 g/mol). Yield: (0.24 g, 93%). ^1^H NMR (400 MHz; DMSO-d_6_): 7.56 (6H, d, J = 8.4 Hz, CH-Asp), 7.47 (6H, d, J = 7.6 Hz, CH-Asp), 7.39 (12H, s, CH-Asp), 7.33 (27H, d, J = 7.6 Hz, uncomplexed Ar-H), 7.26 (24H, d, J = 8.4 Hz, uncomplexed Ar-H), 6.28 (24H, s, complexed Ar-H), 5.23 (30H, s, Cp-H), 5.12 (12H, s, CH_2_-O), 2.21 (12H, s, CH_2_), 2.08 (18H, s, Asp-CH_3_), 1.66 (9H, s, CH_3_). ^13^C NMR δ_c_ (100 MHz; DMSO-d_6_,): 170.60 and 169.65 (CO), 153.87, 153.52, 152.12, 146.59, 134.74, and 130.52 (quat-C), 133.80, 132.65, 129.76, 129.62, 129.09, 121.12, 120.96, 120.36, and 118.33 (uncomplexed Ar-C), 130.98, 130.66, 126.84, 125.80, 124.62, and 122.23 (Asp-Ar-C) 78.40 (Cp-C), 75.60 and 75.49 (complexed Ar-C), 65.46 (ArCH_2_-O), 43.20, 33.81, 32.06, and 31.64 (CH_2_), 27.58 (CH_3_), 21.19 (Asp-CH_3_). ATR-FTIR; ν_max_/cm^-^1: 2926 (Ar-CH), 2850 (Cp-CH), 1731 (CO), 1226 (C-O-C). Elemental analysis for C_219_H_180_O_42_Fe_6_P_6_F_36_: calculated %C 56.10, %H 3.89, and found %C 56.62, and %H 4.10.

### 3.3. Aspirin-Terminated G2-D6-Asp Dendrimer

Aspirin was paired with a second-generation dendrimer by following the Steglich esterification reaction of aspirin and hydroxyl-terminated dendrimer. A 25 mL round-bottom flask was charged with **G2-D5** (0.25 g, 0.02 mmol), **Aspirin** (0.15 g, 0.82 mmol), DMAP (0.18 g, 1.55 mmol), 10 mL of DMF, and then DCC (0.16 g, 0.77 mmol) (Molecular weight 12,847.33 g/mol). Yield: (0.22 g, 92%). ^1^H NMR (400 MHz; DMSO-d_6_): 7.55 (12H, d, J = 7.6 Hz, CH-Asp), 7.47 (12H, d, J = 7.6 Hz, CH-Asp), 7.36 (24H, CH-Asp), 7.33 (75H, d, J = 7.2 Hz, uncomplexed Ar-H), 7.25 (72H, uncomplexed Ar-H), 6.27 (72H, s, complexed Ar-H), 5.23 (90H, s, Cp-H), 5.12 (36H, s, CH_2_-O), 2.21 (36H, s, CH_2_), 2.08 (36H, s, CH_3_-Asp), 1.61 (27H, s, CH_3_). ^13^C NMR δ_c_ (100 MHz; DMSO-d_6_,): 170.72 and 169.83 (CO), 153.87, 153.52, 152.14, 146.61, 134.73, and 130.54 (quat-C), 133.8, 132.65, 129.76, 129.63, 129.07, 120.39, and 118.33 (uncomplexed Ar-C), 130.98, 130.66, 127.21, 125.80, 124.64, and 122.24 (Asp-Ar-C) 78.38 (Cp-C), 75.59 and 75.48 (complexed Ar-C), 65.22 (ArCH_2_-O), 42.46, 42.20, 33.82, 32.08, 31.23, 30.33 (CH_2_), 27.59 (CH_3_), 21.20 (Asp-CH_3_). ATR-FTIR; ν_max_/cm^−1^: 2932 (Ar-CH), 2857 (Cp-CH), 1736 (CO), 1234 (C-O-C). Elemental analysis for C_591_H_492_O_102_Fe_18_P_18_F_108_: calculated %C 55.28, and %H 3.86, and found %C 55.86, %H 4.12. 

### 3.4. Aspirin-Terminated G3-D9-ASP Dendrimer

In a process analogous to the coupling of aspirin with the second-generation dendrimer, aspirin was conjugated to the third-generation dendrimer. A 25 mL round-bottom flask was charged with **G3-D8** (0.25 g, 0.01 mmol), **Aspirin** (0.12 g, 0.66 mmol), DMAP (0.16 g, 1.31 mmol), 10 mL of DMF, and then DCC (0.14 g, 0.67 mmol) (Molecular weight 29139.83g/mol). Yield: (0.21 g, 92%). ^1^H NMR (400 MHz; DMSO-d_6_): 7.55 (24H, d, J = 7.6 Hz, CH-Asp), 7.47 (24H, d, J = 7.6 Hz, CH-Asp), 7.36 (48H, d, J = 5.2 Hz, CH-Asp), 7.33 (171H, d, J = 8.2 Hz, uncomplexed Ar-H), 7.25 (168H, d, J = 7.2 Hz, uncomplexed Ar-H), 6.28 (168H, s, complexed Ar-H), 5.23 (210H, s, Cp-H), 5.12 (84H, s, CH_2_-O), 2.21 (84H, s, CH_2_), 2.08 (72H, s, CH_3_-Asp), 1.61 (63H, s, CH_3_). ^13^C NMR δ_c_ (100 MHz; DMSO-d_6_,): 173.20, 170.73, 169.86, 169.40, and 165.33 (CO), 153.78, 153.52, 153.43, 152.15, 146.98, 146.66, 146.43, 134.71, and 130.56 (quat-C), 133.80, 132.65, 129.66, 129.62, 129.10, 120.98, 120.36, and 118.33 (uncomplexed Ar-C), 130.98, 130.66, 125.80, 123.09, and 121.12 (aspirin Ar-C) 78.38 (Cp-C), 75.59 and 75.49 (complexed Ar-C), 65.41 (ArCH_2_-O), 33.82, 32.06, 31.66, 30.80 and 30.57 (CH_2_), 27.61 (CH_3_), 21.23 (Asp-CH_3_). ATR-FTIR; ν_max_/cm^-^1: 2930 (Ar-CH), 2866 (Cp-CH), 1739 (CO), 1221 (C-O-C). Elemental analysis for C_1335_H_1116_O_222_Fe_42_P_42_F_252_: calculated %C 55.02, and %H 3.76, and found %C 55.68, and %H 3.97.

### 3.5. Aspirin-Terminated G4-D12-Asp Dendrimer

The fourth generation of dendrimer was functionalized with aspirin by following the Steglich esterification reaction of aspirin and hydroxyl-terminated dendrimer. A 25 mL round-bottom flask was charged with **G4-D11** (0.25 g, 0.004 mmol), **Aspirin** (0.11 g, 0.66 mmol), DMAP (0.14 g, 1.31 mmol), 10 mL of DMF, and then DCC (0.10 g, 0.47 mmol) (Molecular weight 61742.39g/mol). Yield: (0.2 g, 90%). ^1^H NMR (400 MHz; DMSO-d_6_): 7.56 (48H, d, J = 8 Hz, CH-aspirin), 7.47 (48H, d, J = 7.6 Hz, CH-aspirin), 7.36 (96H, d, J = 5.4 Hz, CH-aspirin), 7.33 (363H, d, J = 7.4 Hz, uncomplexed Ar-H), 7.25 (360H, d, J = 8.4 Hz, uncomplexed Ar-H), 6.28 (360H, s, complexed Ar-H), 5.23 (450H, s, Cp-H), 5.12 (180H, s, CH_2_-O), 2.21 (180H, s, CH_2_), 2.08 (144H, s, CH_3_-Asp), 1.61 (135H, s, CH_3_). ^13^C NMR δ_c_ (100 MHz; DMSO-d_6_,): 173.19, 170.72, 169.82, 169.39, and 165.30 (CO), 153.87, 153.52, 153.31, 152.20, 152.14, 146.96, 146.61, 146.37, 134.72, and 130.50 (quat-C), 133.80, 132.65, 130.97, 129.63, 129.09, 120.95, 120.36, and 118.33 (uncomplexed Ar-C), 130.66, 125.85, 123.11, and 121.12 (Asp-Ar-C) 78.39 (Cp-C), 75.59 and 75.49 (complexed Ar-C), 65.19 (ArCH_2_-O), 45.54, 33.80, 33.41, 32.04, 31.66, 30.81 and 30.62 (CH_2_), 27.63 (CH_3_), 21.26 (aspirin-CH_3_). ATR-FTIR; ν_max_/cm^-^1: 2934 (Ar-CH), 2868 (Cp-CH), 1740 (CO), 1223 (C-O-C). Elemental analysis for C_2823_H_2364_O_462_Fe_90_P_90_F_540_: calculated %C 54.86 %H 3.83, and found %C 54.68, and %H 4.28.

## 4. Results and Discussion

### 4.1. Syntheses and Characterization of the Aspirin-Based Dendrimers 

**Scheme 1:**
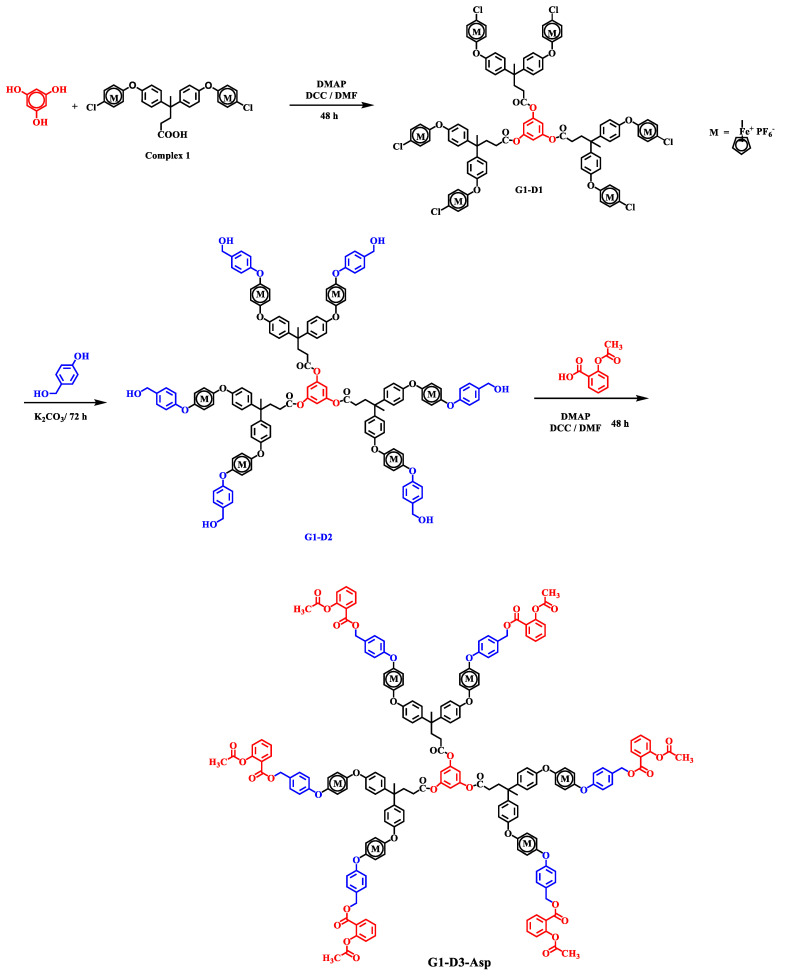
Schematic representation of the synthesis of **G1-D3-Asp.**

**Scheme 2:**
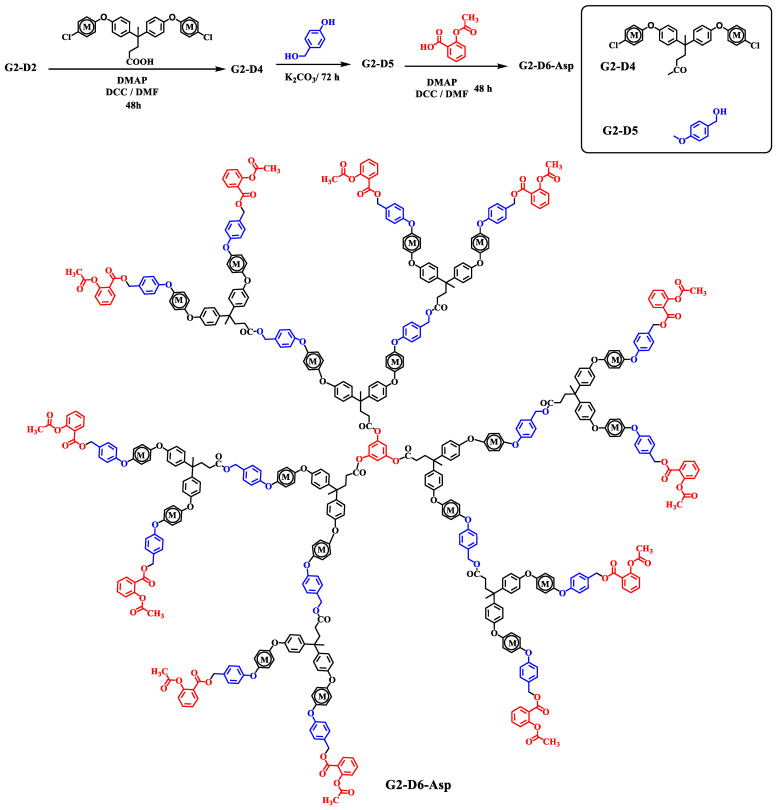
Schematic representation of the synthesis of **G2-D6-Asp.**

**Scheme 3:**
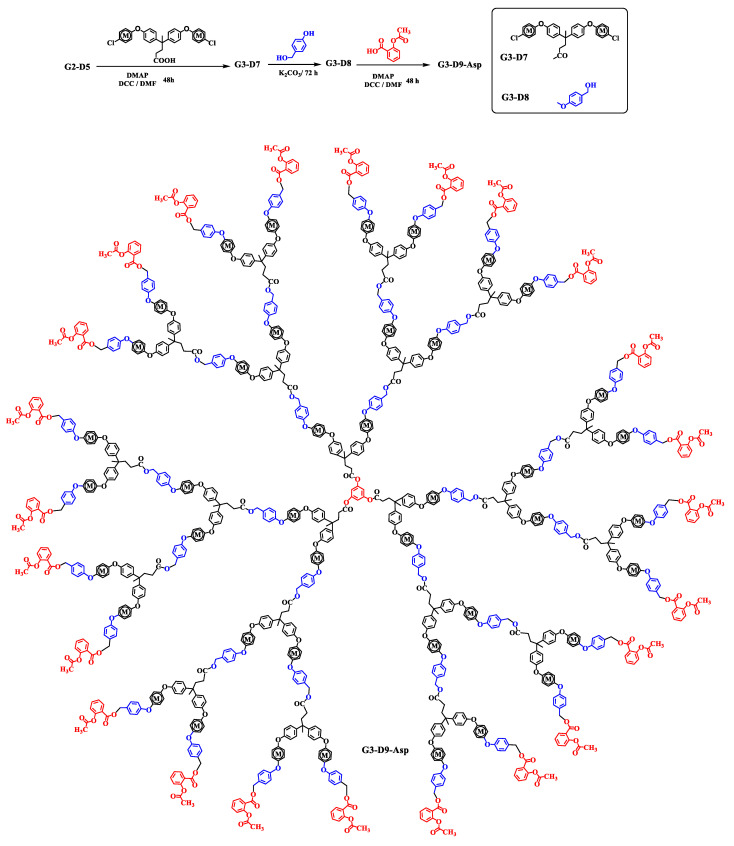
Schematic representation of the synthesis of **G3-D9-Asp.**

**Scheme 4:**
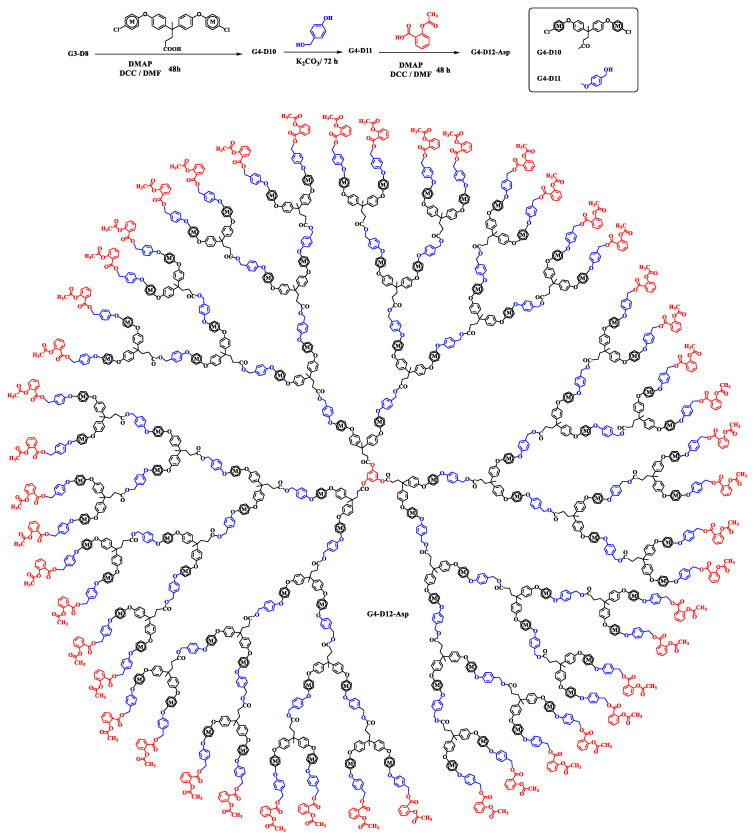
Schematic representation of the synthesis of **G4**-**D12-Asp.**

This article aims to design four generations of dendrimers with aspirin units in the periphery and evaluate these molecules for antimicrobial, anticancer, and anti-inflammatory activities. The divergent synthetic method was utilized to build the dendrimer from the first to fourth generation, and an excellent yield was gained around 90%. Under moderate conditions, the Steglich esterification procedure was used interchangeably with nucleophilic aromatic substitution reactions to build dendrimer generations [6,56]. Coupling aspirin with dendrimer generations was conducted by the Steglich esterification procedure using appropriated molar ratios. As an example, the first-generation aspirin-based dendrimer was obtained by the reaction between **G1-D2** and aspirin by obtaining the appropriate molar ratio yielding **G1-D3-Asp**. Through similar iterative steps, other aspirin-based dendrimers were designed, as shown below (Scheme 1, Scheme 2, Scheme 3 and Scheme 4). 

**Figure 1:**
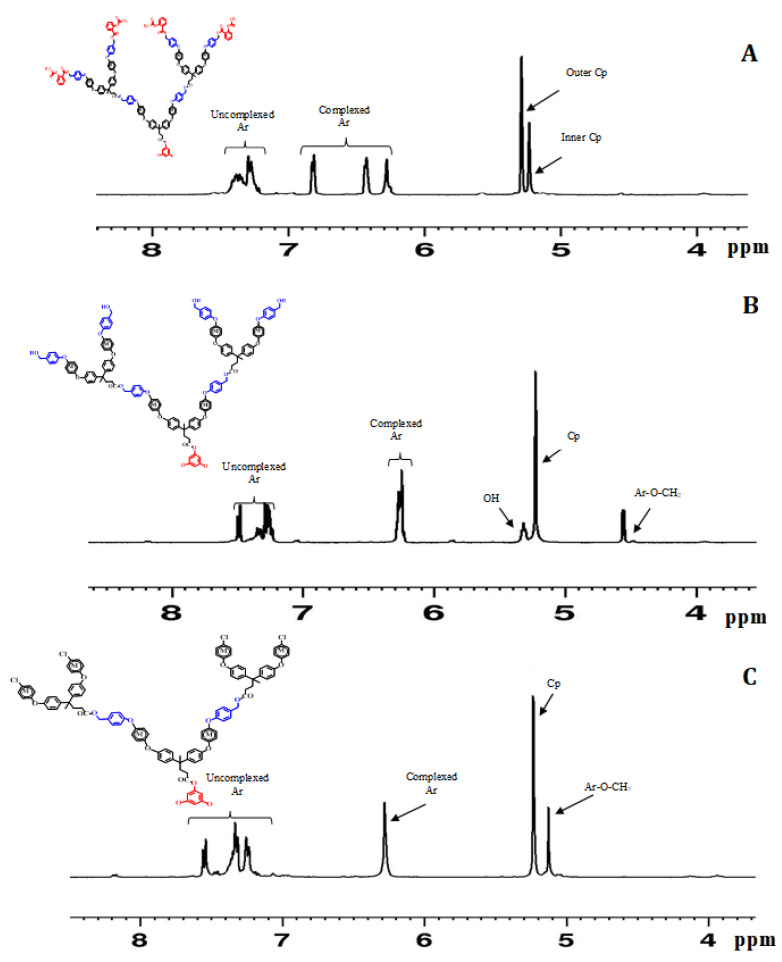
^1^H NMR spectra of (**A**) **G2-D4**, (**B**) **G2-D5**, and (**C**) **G2-D6-Asp** in DMSO-d_6_.

^1^H and ^13^C NMR, FT-IR, TGA, and elemental analysis were used to confirm the capping of aspirin with dendrimer generations. In the ^1^H and ^13^C NMR spectrum, roughly the same trend was observed for the four generations with slight downfield shifting as generation increases. For example, in the ^1^H NMR spectrum of dendrimer **G2-D4**, the complexed aryl protons were observed as three peaks. The protons of the inner complexed aryl groups appeared at 6.30 ppm, evidently differentiated from those of the outer complexed aryl protons, which resonated at two different frequencies, 6.43 and 6.82 ppm, due to non-equivalent attached groups. On peripheral functionalization of **G2-D4** with 4-hydroxy benzyl alcohol to build higher generations, for instance in **G2-D5**, these complexed aryl protons resonated upfield as one peak at δ = 6.28 ppm due to equivalent surrounding etheric oxygen groups. In addition, in **G2-D4**, two peaks appeared at 5.28 and 5.22 ppm, corresponding to the two distinct Cp protons. These Cp peaks showed integration in agreement with the ratio of Cp protons pendent to the chloro-arenes in the periphery to those in the inner arenes with the etheric bridges. There were two Cp peaks of **G2-D4** due to the different environments, which became one peak in **G2-D5** due to the surrounding all-cyclopentadienyl groups with etheric oxygen at the *para* positions. This peak still appeared as a singlet in **G2-D6-Asp** for the same reason, with a negligible shift. Moreover, in the dendrimer **G2-D5**, a singlet peak at δ = 4.58 ppm was observed. This peak referred to the 24 protons of methylene groups in 4-hydroxy benzyl alcohol. Following the periphery functionalization of the dendrimer **G2-D5** with aspirin moieties to yield **G2-D6-Asp**, these protons resonated downfield at δ = 5.12 ppm. The disappearance of the OH peak of hydroxy benzyl alcohol in the ^1^H NMR spectrum of **G2-D6-Asp** is another indication of a successful esterification reaction between **G2-D5** and aspirin, (Figure 1). This peak was resonated at δ = 5.32 ppm in **G2-D5** dendrimer, and due to the formation of the ester group, the peak has been omitted. Additional peaks were also observed in the ^1^H NMR spectrum of **G2-D6-Asp** at δ = 7.56, 7.47, and 7.39 ppm; these peaks indicate the 24 protons of aromatic aspirin. The ^1^H NMR spectrum of higher generations has exhibited similar observations after reacting with aspirin. A new CH_3_ peak also appeared at δ = 2.08, which refers to the Asp-methyl groups. These observations suggested that aspirin was successfully coordinated into the dendrimer generations.

**Figure 2:**
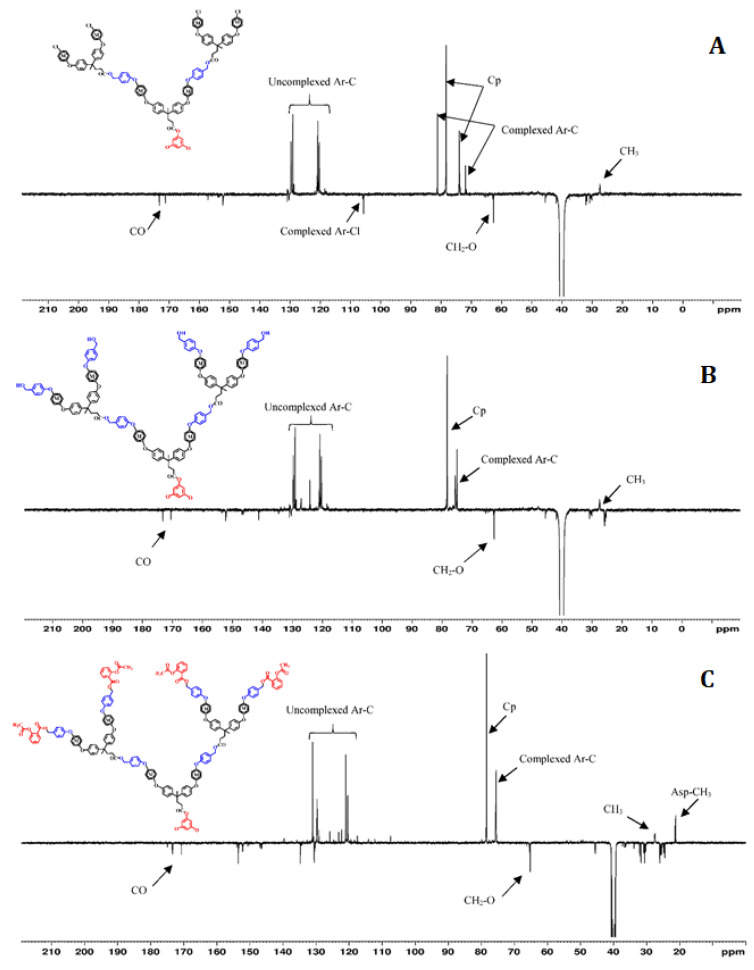
^13^C-NMR spectra of (**A**) **G2-D4**, (**B**) **G2-D5**, and (**C**) **G2-D6-Asp** in DMSO-d_6_.

Successful coupling of aspirin with four generations of dendrimers was also confirmed by using ^13^C NMR spectroscopy. It is important to mention that similar observations were detected in the ^13^C NMR spectra of the four generations after conjugation with aspirin, which could be due to equivalent environments. For example, carbonyl groups were observed as two peaks around 170.60 and 169.65 ppm in the four generations. These peaks referred to the ester linkage of generations and the ester linkage of the core, respectively. The Cp carbons were observed as two peaks around 79.68 and 76.89 ppm in **G2-D4**, one peak at 76.89 ppm which refers to the inner Cp carbon, while the peak around 79.68 ppm refers to the outer Cp carbon. These Cp carbons were slightly shifted up field as a single peak at 80.12 ppm in G2-D5 due to the equivalent environments. In addition, one peak corresponding to the Cp carbons around 78.40 ppm appeared for all aspirin-based dendrimers, (Figure 2).

Furthermore, the complexed carbons of outer and inner aril groups appeared as two peaks around 87.98 and 74.76 ppm in **G2-D4,** respectively. These peaks were observed around 75.67 and 76.10 ppm in **G2-D5.** In the presence of the peripheral aspirin moieties, the complexed carbons vibrated around 75.60 ppm, while the uncomplexed carbons were detected between 130.98 and 118.33 ppm. In addition, the carbon of ArCH_2_O was found around 65.20 ppm, the quaternary carbons were noticed between 153.87 and 130.50 ppm, and aspirin-CH_3_ carbons were located around 27.58 ppm in four generations.

The ATR-FTIR absorption spectra of prepared dendrimers exhibited the presence of characteristic (Ar-CH), (Cp-CH), (CO), and (C-O-C) bands, around 2926, 2850, 1731, and 1226 cm^−1^, respectively. Elemental analysis has further confirmed the dendrimers’ formation, as outlined in the experimental section.

### 4.2. Morphological Characterization

**Figure 3:**
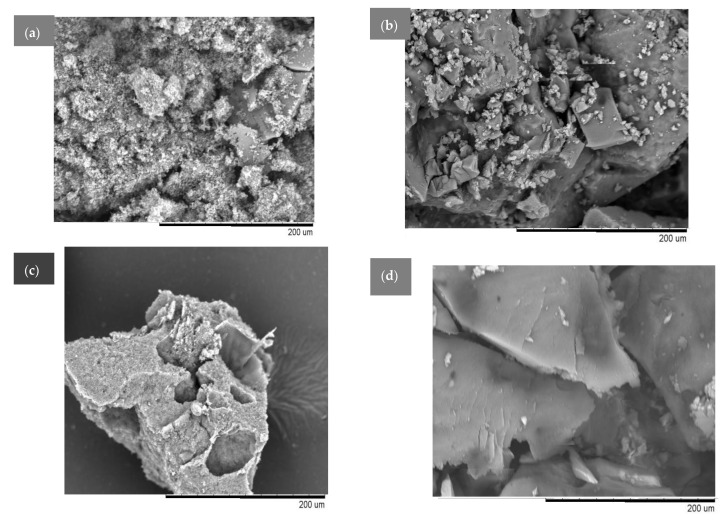
Scanning Electron Microscope Images for **G1-D3-Asp** (**a**), **G2-D6-Asp** (**b**), **G3-D9-Asp** (**c**), **G4-D12-Asp** (**d**).

The surface texture was examined as presented in a powder film for the prepared dendrimers by scanning electron microscopy (SEM). Representative surface morphologies of the film are presented in (Figure 3). The **G1-D3-Asp** had no specified shape and appeared as globular irregular amorphous (Figure 3a). The micrographs of **G2-D6-Asp** indicated the residue consisted of an agglomeration of different particle sizes and shapes. The large substrate particles were of different sizes with sharp edges, surrounded with irregular amorphous particles (Figure 3b). The morphology began to exhibit a rock-like agglomeration of different sizes and shapes. Particle agglomeration was also observed with the **G3-D9-Asp**, with particles having a rock-like appearance with different pore sizes (Figure 3c). Well-defined particles were observed in **G4-D12-Asp** residues, with particles having thin leaf-like slice shapes with sharp edges and almost uniform in size (Figure 3d).

### 4.3. Thermal Analysis of Synthesized Dendrimers

**Figure 4:**
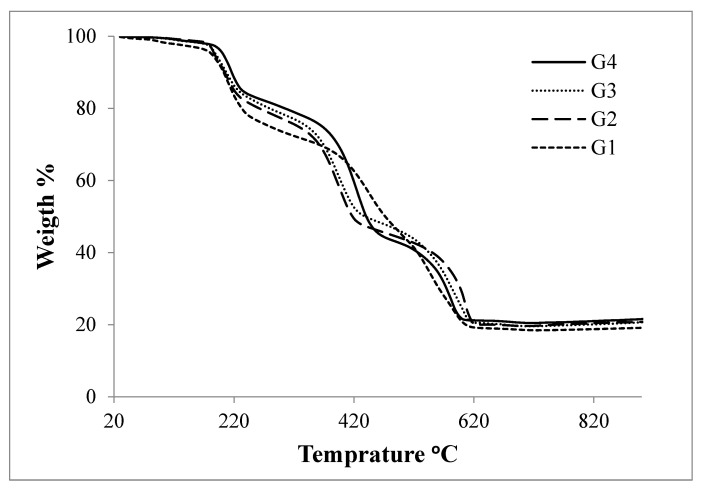
Thermogravimetric analysis (TGA) of G1-D3-Asp, G2-D6-Asp, G3-D9-Asp, and G4-D12-Asp.

The thermal stability of the dendrimers was studied using thermogravimetric analysis (TGA) under nitrogen. The prepared dendrimers containing cationic cyclopentadienyliron in their branches showed similar three characteristic decomposition stages at slightly different temperatures (Figure 4), for example, 24% weight loss at 150 °C for **G1-D3-Asp**, 19% weight loss at 178 °C for **G2-D6-Asp**, 18% weight loss at 180 °C for **G3-D9-Asp**, and 18% weight loss at 200 °C for **G4-D12-Asp.**

The first degradation indicates the loss of the attached cyclopentadienyliron moieties from the backbones of these dendrimers. Further significant weight losses were exhibited between 250 °C and 380 °C, which were attributed to the second decomposition. The second decomposition was recorded around 250 °C for **G1-D3-Asp** with 26% weight loss, 340 °C for **G2-D6-Asp** with 34% weight loss, 360 °C for **G3-D9-Asp** with 32% weight loss, and 380 °C for **G4-D12-Asp** with 37% weight loss.

The third mass losses between 456 °C and 560 °C were assigned to thermal decomposition, oxidation, and volatilization of the remaining dendrimer backbone, recorded around 456 °C for **G1-D3-Asp** with 30% weight loss, 560 °C for **G2-D6-Asp** with 25% weight loss, 540 °C for **G3-D9-Asp** with 28% weight loss, and 530 °C for **G4-D12-Asp** with 20% weight loss. In the last part of these analyses, which was around 620 °C, the remaining content was around 20% for four generations assigned to iron’s residual weight measured. [52,55].

### 4.4. Electrochemical Properties

**Figure 5:**
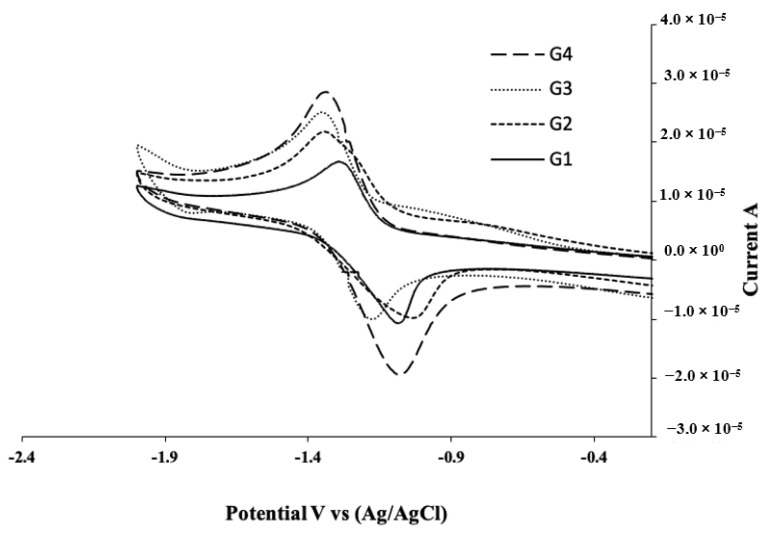
Representative cyclic voltammogram for **G1-D3-Asp**, **G2-D6-Asp**, **G3-D9-Asp**, **G4-D12-Asp** in 0.1 M Bu_4_NPF_6_ in propylene carbonate.

Electrochemical properties were investigated for produced dendrimer generations at 25 °C and 0 °C in 0.1 M Bu_4_NPF_6_ solution using a propylene carbonate (as a supporting electrolyte), a Pt wire counter electrode, an Ag/Ag^+^ reference electrode, and a glassy carbon working electrode. The potential value was scanned in the range of 0 to −2.0 mV/s. The synthetic method allowed the association of redox-active η6-aryl-η5 cyclopentadienyl iron (II) centers in the dendritic branches at every repeated synthetic step to form layers of redox centers, which are understood to be redox-active. All aspirin-based dendrimer complexes showed a single reversible redox wave, with different intensities dependent on dendrimer generation, and the average *E*_1/2_ value was between −1.18 V and −1.26 V (Figure 5). The presence of a single redox wave was expected due to the noninteracting and equivalent redox centers. It is worth stating that increasing the number of cationic iron centers in successive dendrimeric generations enhanced the wave’s intensity. The electron transfer rate between the electrodes and iron centers in different dendritic generations prevented the splitting of the reduction wave and overlapped cathodic currents, resulting from the reduction of iron centers [52,53].

### 4.5. Antimicrobial Activity

Many studies have confirmed that NSAIDs display antibacterial properties, but the mechanism of action is not clear yet [6,63,64,65]. Aspirin and ibuprofen showed antibacterial activity toward some types of pathogenic bacteria [66]. With a modification of aspirin in some derivatives, the gastrointestinal toxicity of aspirin has decreased, and the pharmacological properties have been improved [39,66]. Anti-fungal activity was also found to be a property of certain aspirin derivatives [43].

Agar well diffusion by using nutrient agar medium was used to examine the antimicrobial activity of the organoiron dendrimers **G1-G4.** The inhibition zones in mm for the four generations of aspirin-based organoiron dendrimers were determined against two Gram positive bacteria (*B. subtills* and *S. aureus*), two Gram-negative bacteria (*E. coli* and *K. pneumoniae*), and two yeasts *C. albicans* and *A. niger*, employing the modified Kirby–Bauer disc diffusion method. Control experiments against each strain were carried out with known antimicrobial agents’ ampicillin, gentamicin, and nystatin for the three types, respectively. The solvent control was DMSO, and the concentration of the organoiron dendrimers was tested against both bacterial and fungal strains was 15 mg/mL.

**Figure 6:**
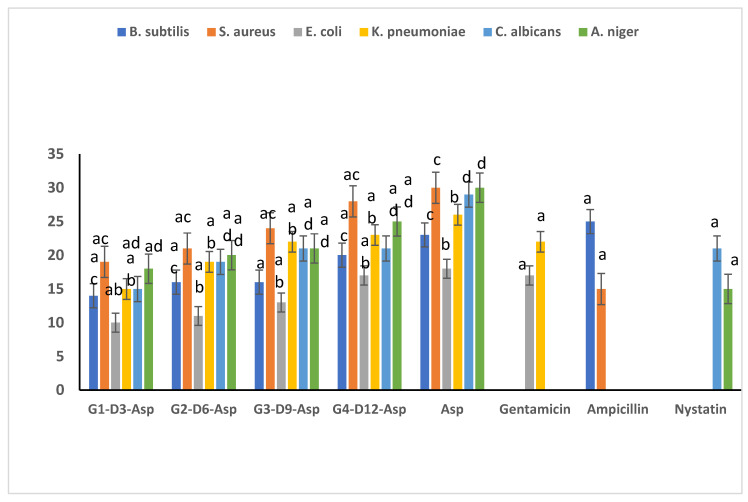
The results of antimicrobial activity for the aspirin and the **G1**, **G2**, **G3** and **G4** dendrimers at 15 mg/mL against different strains of bacteria and fungi compared to references. All data are represented as mean ± SE. Statistical significance was measured at *p* < 0.05 based on one-way analysis of variance ANOVA test followed by Tukey’s test for multiple comparisons. **a** was significantly different from the aspirin group at *p* < 0.05, **b** was significantly different from the gentamicin group at *p* < 0.05, **c** was significantly different from the ampicillin group at *p* < 0.05, and **d** was significantly different from the nystatin group at *p* < 0.05.

The results of inhibition exhibited excellent activity against all bacterial and fungi strains in addition to the anti-inflammatory effect due to the presence of aspirin moieties. The fourth generation **G4** dendrimer showed notable antimicrobial activity, and its inhibition zone for all Gram-positive and Gram-negative bacteria and fungus are higher than that of the standard drugs. The antimicrobial activity of the four aspirin-based organoiron dendrimers increased by increasing the generation of the dendrimers for most types of microorganisms, determined by the inhibition zones in mm (Table 1) (Figure 6). Comparing the antibacterial activity of the organoiron complexes that were earlier reported [67], we can undoubtedly see the enhancement of the activity for the organoiron dendrimers, especially the dendrimers containing aspirin.

The association of individual units of aspirin into self-assembled highly ordered dendrimers gave excellent properties to the newly synthesized dendrimers. The essential reason for this is the possibility of highly functionalized dendrimers with an efficient material, aspirin, that can be useful in biomedical applications. This has opened new avenues for their use in material science and bioscience delivery applications. 

For instance, notable antibacterial activity was shown against *B. subtilis* for the first generation **G1-D3-Asp**, having 6 aspirin units at the periphery, while the best result was found for the fourth generation **G4-D12-Asp**, containing 48 aspirin units at the periphery. This is likely due to the increase in the total outside aspirin molecules of the dendrimer. Increasing the number of available drug groups can enhance the interaction with the targeted microorganism. All aspirin-based dendrimers showed a significant increase in the antibacterial activity against *S. aureus*, comparable with the reference drug ampicillin; however, **G4**-**D12-Asp** showed the most potent antibacterial activity. 

Gram-negative bacteria are the reason for many hospital-acquired infections, and they are highly resistant to most antibiotic drugs, likely due to the presence of an outer membrane structure that is absent in Gram-positive bacteria [68]. This study reveals for the first time that organoiron dendrimers linked with aspirin exhibit an excellent Gram-negative activity comparable to the reference gentamicin. 

Increasing the number of aspirin units increases the action on the pathogenic microbes. Dendrimers in lower generations have minor activity against Gram-negative bacteria *E. coli* and *K. pneumoniae*. Similar activity was observed against *E. coli* and *K. pneumoniae* for both **G4-D12-Asp** and the reference drug. 

Aspirin is proven to have an anti-fungal effect, however, its effect on candida biofilm is not completely understood [69]. Some studies have confirmed that treating abiotic surfaces with aspirin is successful in annihilating biofilms of *C. albicans*, *E. coli,* and *P. aeruginosa* [43,70,71]. The anti-fungal activities of the tested dendrimers were determined against *C. albicans* and *A. niger.* It has been found that all generations **G1-G4** were exhibited greater anti-fungal activity at the tested concentration. From the result, **G3-D9-Asp** and **G4**-**D12-Asp** at a concentration of 15 mg/mL for 24 h were required to annihilate the *C. albicans* biofilm to the same level as the reference drug nystatin, while **G1** and **G2** were a bit lower. As in the antibacterial study, the increase in the number of aspirin units results in increased activity. The inhibition zone of the *A. niger*, however, showed higher effect of all aspirin-based dendrimers, at the same concentration, than nystatin. Pure aspirin showed the best result against *C. albicans* biofilm and *A. niger*. The use of aspirin therefore may have the advantage of preventing the development of drug resistance in the future.

**Figure 7:**
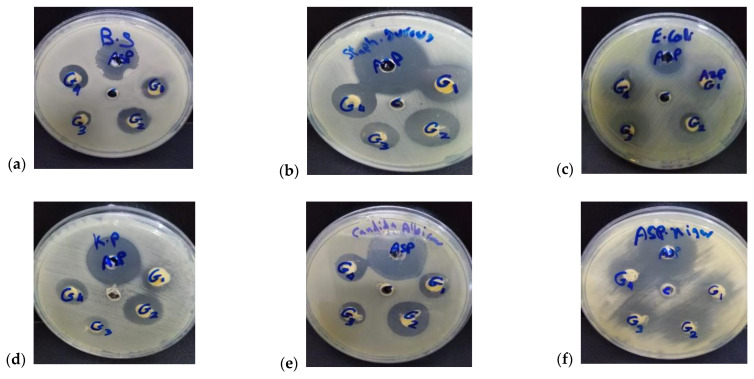
Images of Petri dishes with the inhibition zones against gram-positive bacteria, (**a**) *B. subtilis*; (**b**) *S. aureus*; two Gram-negative bacteria, (**c**) *E. coli;* and (**d**) *K. pneumoniae*, and two yeast, (**e**) *C.* albicans, and (**f**) *A. niger*, of aspirin and its derivatives **G1-D3-Asp**, **G2-D6-Asp**, **G3-D9-Asp**, and **G4-D12-Asp**.

It has been reported that the organoiron macromolecular complexes enhance antimicrobial activity via the induction of cellular oxidative stress, as previously mentioned [72,73]. Due to the oxidation-reduction activities, they are likely to generate reactive oxygen species (ROS) via electron transfer to oxygen [72,73]. The presence of ROS influences oxidative strain, a cellular protection plan employed against a broad spectrum of microbes [6,8,57,74]. By increasing the number of the iron centers η6-arene–η5-cyclopentadienyliron (II) complex through the dendritic branches, the efficiency of the dendrimers against these tested types of bacteria and fungi will be improved. The sample of the images of Petri dishes utilized to measure inhibition zone areas of the dendrimers from **G1** to **G4** and aspirin with the tested bacteria and fungi are presented in Figure 7.

Although the free drug Aspirin had higher antibacterial and antifungal activity than most of the dendrimer generations, we found the fourth generation has also a slight increase in the effect compared to the reference drug. The reduction in activity compared to aspirin itself could be due to the decrease in the solubility of the fourth-generation dendrimer. Solubility is one of the challenges that still needs to be addressed in order to make dendrimers a good choice for pharmaceutical researchers in the future.

### 4.6. Anticancer Activity

**Figure 8:**
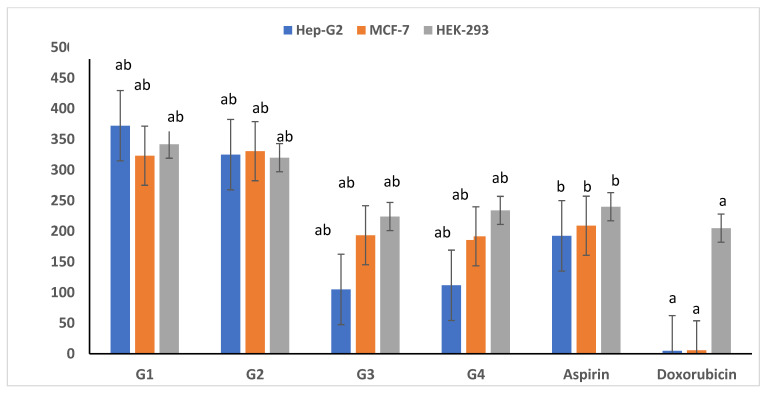
The results of cytotoxicity activity for the aspirin and **G1-D3-Asp**, **G2-D6-Asp**, **G3-D9-Asp**, and **G4**-**D12-Asp** at 15 mg/mL against Hep-G2, MCF-7, and HEK-293 compared to doxorubicin. All data are represented as mean ± SE. Statistical significance was measured at *p* < 0.05 based on one-way analysis of variance ANOVA test followed by Tukey’s test for multiple comparisons. **a** significantly different from the aspirin group at *p* < 0.05, and **b** significantly different from the doxorubicin group at *p* < 0.05.

Several studies suggest that non-steroidal anti-inflammatory drugs (NSAIDs), such as aspirin, that inhibit prostaglandin (PGE2) production could be used as a protecting drug against formation of tumors and may play a role during tumor progression [34,75]. Furthermore, it has been proven that low-dose aspirin inhibits tumor production and that the aspirin antitumor effect is stronger when treatment begins before tumor initiation [75]. Aspirin and four generations of aspirin-based dendrimers were tested for their anticancer activity against breast cancer cell lines (MCF-7), hepatocellular cell lines (Hep-G2), and embryonic kidney non cancer cells (HEK-293) (Table 2) (Figure 8). 

Control experiments against each strain were carried out using the well-known anticancer agent doxorubicin. Both **G3-D9-Asp** and **G4-D12-Asp** exhibited notable activity against both cell lines. **G3-D9-Asp** and **G4-D12-Asp** were more effective than aspirin for both (Hep-G2) and (MCF-7). Increasing the number of generations of the dendrimers also resulted in an increase in efficacy against the cell lines. More importantly, the four prepared generations of aspirin-based dendrimers are highly specific towards cancer cell lines only, as they exhibited mild toxicity against the normal cell line (HEK-293), as illustrated in Figure 8.

The functional effects of aspirin as a drug partially depend on cyclooxygenase (COX) inhibition; unlike other NSAIDs, the effect of aspirin by this mechanism is irreversible [75,76]. A recent study showed that the interaction of redox-active center with intracellular biological molecules may be the cause of the ROS formation, which is responsible for oxidative DNA cleavage, and is most likely to be associated with programmed cell death [76,77]. Cyclic voltammetric study of the aspirin-based organoiron dendrimers has revealed that these complexes undergo easy reduction. Thus, inside cells, iron can go from one oxidation state to another under physiological conditions. This redox cycle is also responsible for the toxic ability of iron toward cancer cells, which is accomplished through the stimulation of reactive oxygen species (ROS) formation [78].

### 4.7. Aspirin-Based Dendrimer Inhibits Proliferation of MCF-7 and Hep-G2 Cells

**Figure 9:**
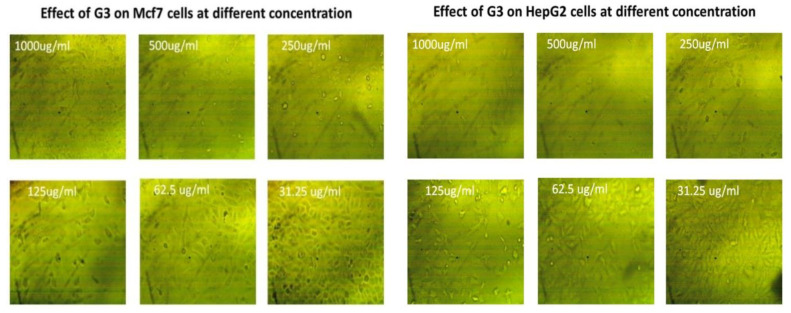
The morphologies of MCF-7 cells (**left**) and HepG2 cells (**Right**) were treated with **G3-D9-Asp** for 72 h. Images were acquired using a microscope (200× magnification).

To examine whether the aspirin-based dendrimers reduced cell viability in metastatic breast cancer MCF-7 and hepatocellular carcinoma Hep-G2, the MTS assay was utilized (Figure 9). MTS assay was performed to confirm the cytotoxic effect of prepared aspirin-based dendrimer complexes. The assay was done according to the published method [79].

It has been demonstrated that aspirin inhibits the human HCC cells and tumor cells by suppressing cell cycle-related molecules and inducing cell apoptosis by increasing oxidative stress [80,81]. Recent analysis has indicated that there was a significant inverse association between aspirin dose and cell proliferation [81,82]. As shown in Figure 9, **G3-D9-Asp** inhibited the cell proliferation in a concentration-dependent manner. Effect of **G3-D9-Asp** on breast cancer cells (MCF-7) showed complete detachment at 1000 µg while it caused partial detachment with circular and shrinkage of cells as cytopathic effect (CPE) at 500 and 250 µg. The treatment exhibited significant abnormal morphological changes with small colonies at 125 µg and slightly morphological changes at 62.5 µg. Indeed, **G3-D9-Asp** was highly efficient in inhibiting liver cancer (HepG2) cell proliferation. It exhibited complete detachment at concentrations of 1000 µg, 500 µg, and 250 µg (Figure 9), while resulting in partial detachment with shrinkage of cells as cytopathic effect (CPE) at 125 µg. A slight morphological change in cells appeared at 62.5 µg, and at the lowest concentration of 31.25, the **G3-D9-Asp** inhibitor showed no toxicity for both cell lines.

### 4.8. In Vitro COX-1 and COX-2 Inhibition Assay

Recent studies on COX-1 and COX-2 inhibitors are focused on increasing the selectivity index of drugs towards COX-1 and COX-2 as it acts as the main effector in the mechanism of pain and inflammation [83,84]. In this paper, all aspirin-based dendrimer complexes were screened for their in vitro cyclooxygenase (COX-1 & COX-2) inhibitory activity compared to the positive drugs aspirin, diclofenac sodium, indomethacin, rofecoxib, and celecoxib using an ovine COX-1/COX-2 assay kit. Studying the data (Table 3), no notable difference was found between the newly prepared organoiron dendrimers and the existing drug aspirin. All aspirin-based dendrimers are good inhibitors of COX-1 and COX-2; however, **G4**-**D12-Asp** exhibited potent in vitro COX-1 inhibitory activity (IC_50_ = 1.2 μM), which is lower than aspirin (IC_50_ = 1.3 μM) with a SI towards COX-2 of 0.20. On the other hand, **G1-D3-Asp** had the highest IC_50_ value for COX-1 between all aspirin-based dendrimers complexes (IC_50_ = 1.5 μM). Second generation **G2-D6-Asp** and third generation **G3-D9-Asp** dendrimers showed similar inhibitory activity of COX-1 (IC_50_ = 1.3 μM), like aspirin. 

The tested generations **G1**, **G2, G3,** and **G4** showed inhibitory activity of the COX-2 enzyme which suggests a potential anti-inflammatory effect. **G4-D12-Asp** showed the best inhibitory activity for COX-2 with IC_50_ of 6.3 μM however, it had the least SI (0.19) towards COX-2, whereas **G1**-**D3-Asp** had the lowest inhibitory activity of COX-1 with an IC_50_ of 6.8 μM and a SI identical to aspirin (0.22). Studying the structure relationship based on the received biological data, the best efficacy compared to the standard drug aspirin was recognized as **G4**-**D12-Asp**. Raising the number of aspirin terminal units in the fourth-generation dendrimer could be the main reason for the inhibitory activity.

### 4.9. Assessment of In Vivo Anti-Inflammatory Activity

#### 4.9.1. In Vivo Rat Paw Edema Assay

The anti-inflammatory activity of the four organoiron dendrimers and aspirin was further evaluated in vivo by carrageenan-induced rat paw edema assay. The paw edema was induced via subcutaneous carrageenan injection in rat paws, and the results were compared to aspirin as a reference drug. The strength of the tested dendrimers to inhibit edema (% edema inhibition) was calculated based on the changes in paw thickness (edema diameter, mm) at different periods; 1 h, 2 h, 4 h, and 6 h (Table 4).

**Figure 10:**
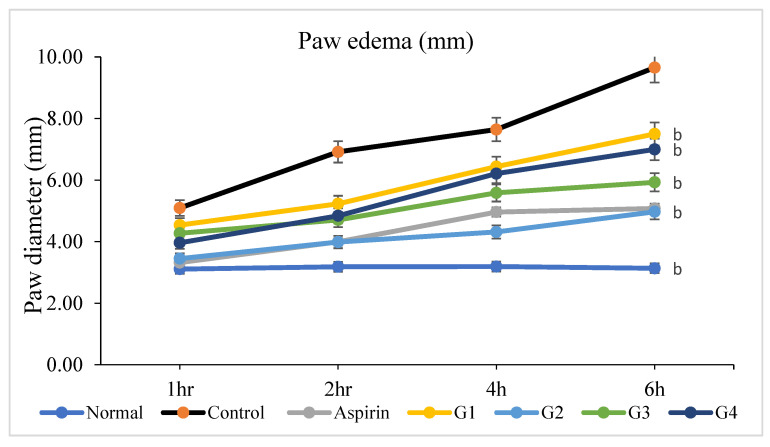
The effect of different treatments on carrageenan-induced paw edema volume at different intervals. All data of the paw edema are represented as mean ± SD. Statistical significance was measured at *p* < 0.05 based on one-way analysis of variance ANOVA test followed by Tukey’s test for multiple comparisons. **b** was significantly different from the control group at *p* < 0.05.

**Figure 11:**
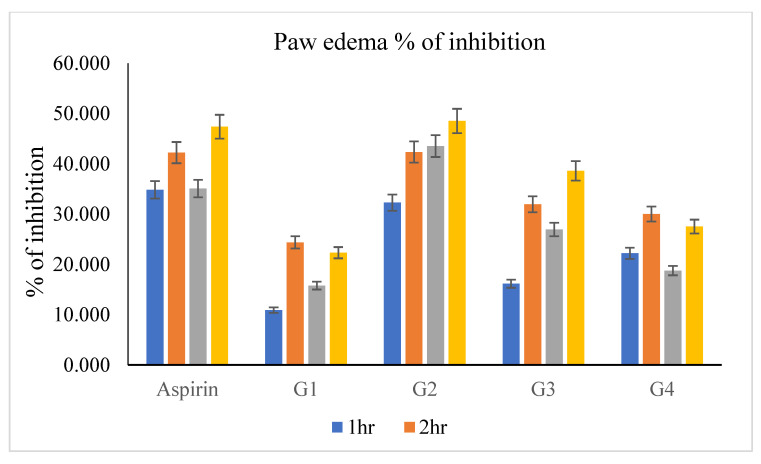
The effect of different treatments on percentage inhibition of edema in carrageenan-induced paw edema.

The tested dendrimers inhibited the development of edema after various measurements and showed notable anti-inflammatory activity (Figure 10). Investigating results in (Table 4) proved that the effect was most obvious when using G2-D6-Asp as it exhibited inhibition by 43.53% after 4 h. This was more efficient as an anti-inflammatory agent than the reference drug aspirin, which exhibited only 35.08% inhibition after the same period. In addition, the same generation showed the highest inhibition between all test dendrimers after 6 h with 48.51%. On the other hand, the first generation G1-D3-Asp dendrimer showed the least anti-inflammatory impact as it exhibited only 22.31% after 6 h, (Figure 11). By increasing the number of aspirin units in the dendrimer from G1-D3-Asp to G2-D6-Asp, the inhibition percentage increased due to the doubled number of aspirin units. When the drug-dendrimer complex enters the body, the hydrolytic cleavage of the covalent bonds between the dendrimer and the drugs will happen chemically or enzymatically, and the drug will be released [6,85,86].

The rest of the tested dendrimers **G3-D9-Asp** and **G4-D12-Asp** displayed moderate anti-inflammatory activity with percentage edema inhibition of 16.15% and 22.20% at 1 h and 38.60 and 27.52% at 6 h, respectively. The reason for the decreased efficiency of the higher generation is the high molecular weight of these dendrimers that causes difficulty getting across the cell membranes and therefore decreasing the bioavailability of the aspirin-based dendrimer complexes. The gained data was compatible with the previous study that outlined that the anti-inflammatory activity of the commercial drug ibuprofen was improved through the attachment to the second generation organoiron dendrimer [6]. 

#### 4.9.2. Determination of Rat Serum PGE2

**Figure 12:**
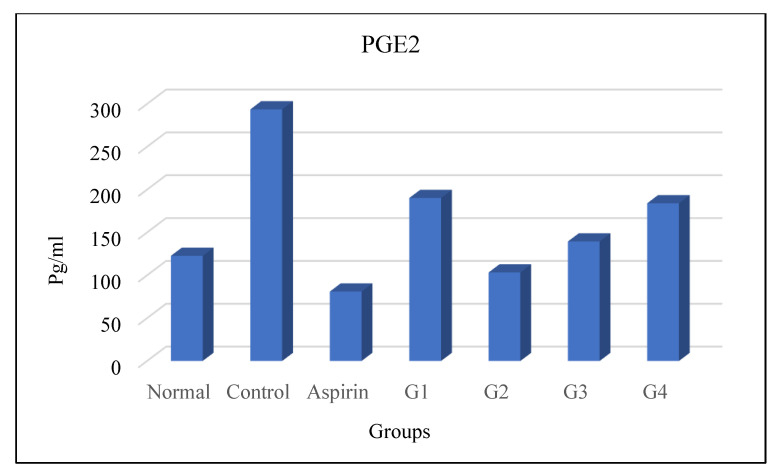
The effect of the tested dendrimers on serum levels of PGE2 in rats.

Several studies reported PGE2 inhibition as one of the most effective methods for the treatment of inflammation. A high level of the potent inflammatory mediator PGE2 is one of the inflammation symptoms. Aspirin-based dendrimer complexes were evaluated for their in vivo serum levels of PGE2. The ability of the dendrimers to inhibit PGE2 was evaluated by measuring the rat serum concentration of PGE2 in blood samples collected after 6 h from subcutaneous carrageenan injection. Percentage PGE2 inhibition was calculated and illustrated in Table 5. Results recorded that all tested four dendrimers provided a good reduction in serum PGE2 (% inhibition = 35.23–64.83) comparable to the reference drug aspirin (% PGE2 inhibition = 72.40). **G2**-**D6-Asp** was the most active complex in this assay with a PGE2 percentage inhibition of 64.83%; the same dendrimer was the most selective towards COX-1 and COX-2 inhibition (Figure 12).

#### 4.9.3. Evaluation of Gastrointestinal Toxicity of the Tested Dendrimers

**Figure 13:**
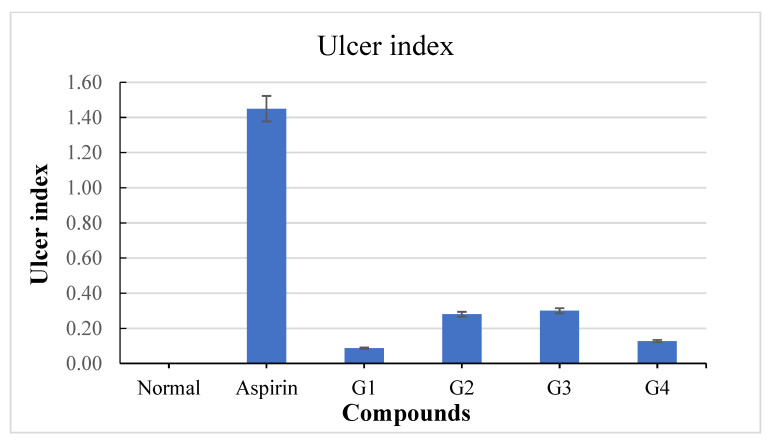
The ulcer index of the tested dendrimer.

The gastric mucosal damage was induced by aspirin and the four tested aspirin-based dendrimers with different damage scores and indexes. Histological examination of the gastric mucosa was described by detecting the ulcer area (mm), ulcer scores, and ulcer indexes. As shown in Table 6 and Figure 13, the synthesized dendrimers significantly decreased damage scores and indexes compared with aspirin, meaning the organoiron dendrimers attached to aspirin are less toxic than aspirin itself on the gastric mucosa. By studying Table 6, we found **G1-D3-Asp** and **G4-D12-Asp**, had the least toxic effect on the gastric mucosa with ulcer area of 5.17 ± 1.13 and 7.58 ± 1.00, respectively and the least ulcer indexes (0.09 ± 0.02 and 0.13 ± 0.02) respectively compared to aspirin with ulcer area of 86.91 ± 9.51 and ulcer index of 1.45 ± 0.16. 

These results give hope for dendrimers to be used in oral drug delivery in the future. Many studies have provided similar results and using dendrimers has started to be approved as safer oral drug delivery [87]. It was found that charged cationic dendrimers commonly acted to reduce potential toxicity in the gastrointestinal tract [87]. By attaching the drug to the surface of dendrimers, it is possible to minimize toxicity and damage of the gastric mucosa while increasing efficiency [88]. 

#### 4.9.4. Histopathology Examination

**Figure 14:**
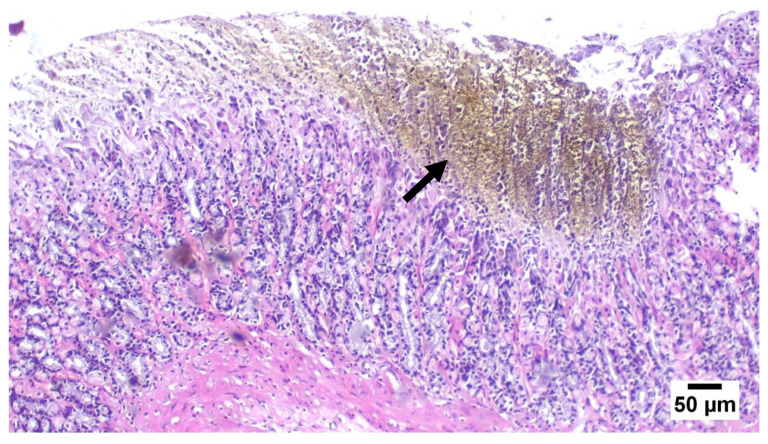
Photomicrograph of stomach, aspirin group, showing extensive mucosal damage, hemorrhage (arrow) (H&E).

**Figure 15:**
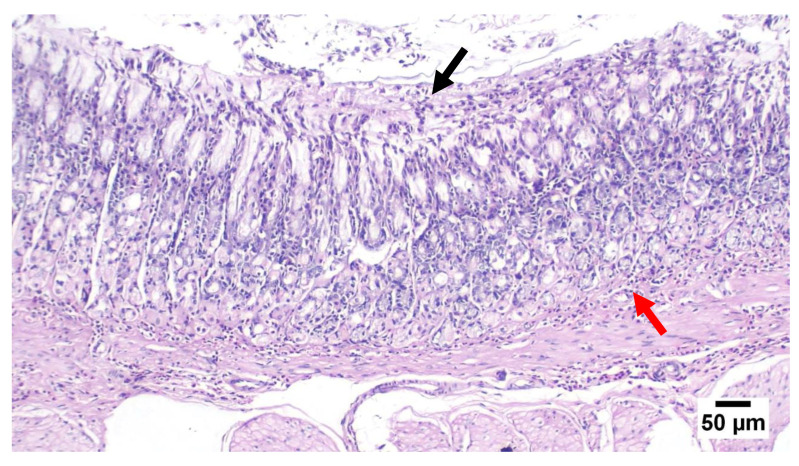
Photomicrograph of stomach, **G1-D3-Asp**, showing surface epithelial loss with exaggerated mucous secretion (black arrow) and mononuclear inflammatory cells infiltration (red arrow) (H&E).

**Figure 16:**
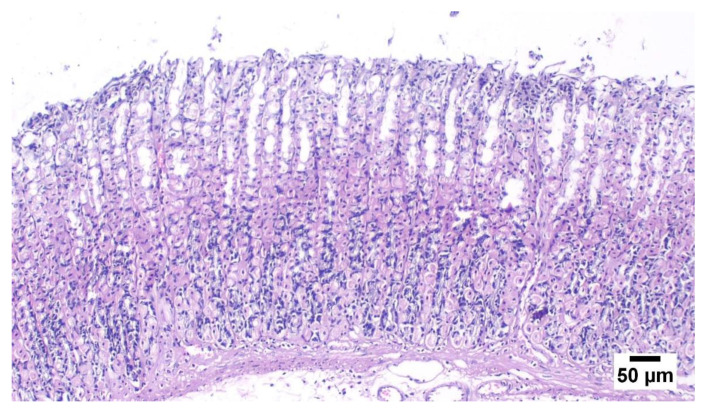
Photomicrograph of stomach, **G2-D6-Asp**, showing mild degeneration in the gastric glands (H&E).

**Figure 17:**
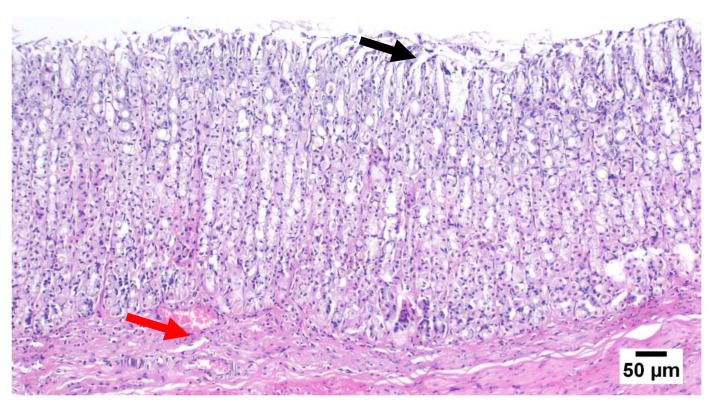
Photomicrograph of stomach, **G3-D9-Asp** showing mild epithelial loss (black loss), congestion (red arrow), and mild inflammatory reaction (H&E).

**Figure 18:**
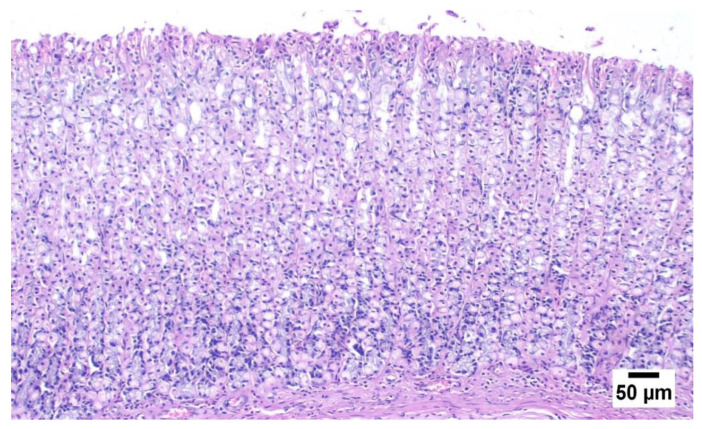
Photomicrograph of stomach, **G4-D12-Asp** showing apparently normal gastric mucosa (H&E).

Recently, many studies have reported the uses of dendrimers as an oral drug delivery system due to their ability to transfer through the epithelial layer of the stomach [89,90]. In order to determine their suitability for oral delivery, we studied the effect of all dendrimers in the gastrointestinal tract compared to aspirin, since anti-inflammatory drugs are the most common cause of gastric ulcers [91]. Microscopic examination of a stomach tissue section from aspirin group (Figure 14) revealed serious histopathological alterations, and extensive mucosal damage was noticed with substantial tissue loss. Severe hemorrhages were observed along with heavy inflammatory cell infiltration. Regarding group 1, **G1-D3-Asp** (Figure 15), the mild epithelial loss was noticed with exaggerated mucus secretion that covered the denuded surface; also, an intense inflammatory reaction was noticed at the deep mucosa. Some other sections showed confined damage to lamina epithelial only. Gastric mucosa of group 2, **G2-D6-Asp** (Figure 16), showed mild epithelial loss and degeneration in the gastric glands with the existence of some cystically dilated glands, and some other sections appeared histologically normal. Group 3, **G3-D9-Asp** (Figure 17), exhibited mild epithelial loss with congestion in the gastric mucosa, and few sections showed a mild inflammatory reaction. No histopathological alterations were observed in group 4, **G4-D12-Asp** (Figure 18), and normal gastric mucosa was observed. Overall our dendrimers showed potential improvements in anti-inflammatory activity and minimizing gastrointestinal toxicity.

**Figure 19:**
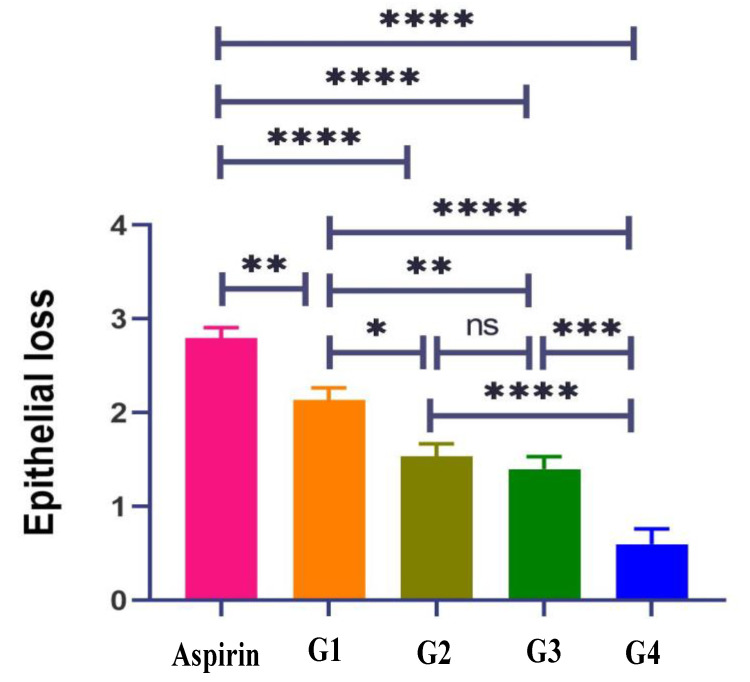
Epithelial loss score. Data were presented as means ± SE. * statistically significant at *p* ≤ 0.1, ** *p* *≤* 0.01, *** *p* ≤ 0.001, **** *p* ≤ 0.0001. Significance was considered at *p* ˂ 0.05. ns-not significant.

**Figure 20:**
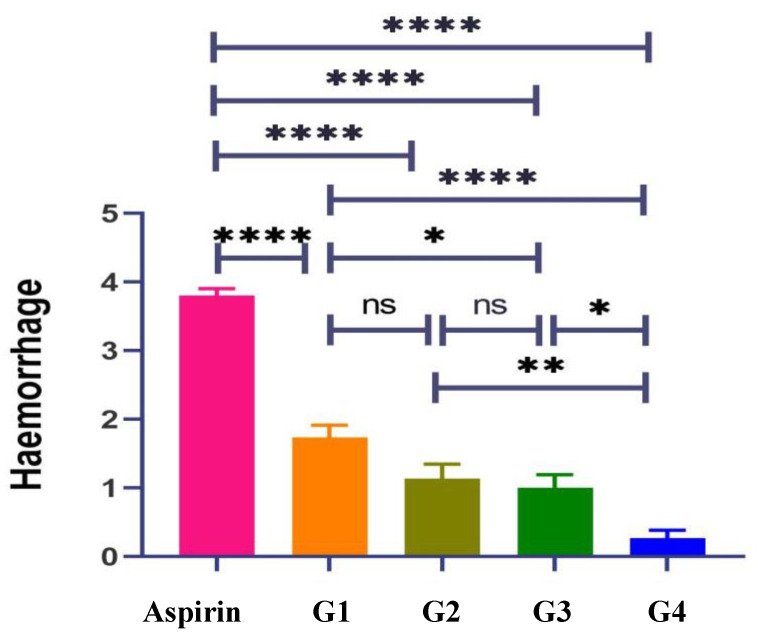
Hemorrhage score. Data were presented as means ± SE. * statistically significant at *p* ≤ 0.1, ** *p* ≤ 0.01, **** *p*≤ 0.0001. Significance was considered at *p* ˂ 0.05. ns-not significant.

Regarding the histologic lesion score, a significantly higher epithelial loss score (Figure 19) was detected in the aspirin group in comparison to the other experimental groups. **G1-D3-Asp** to **G4-D12-Asp** showed significant reduction in epithelial loss when compared to the aspirin group. The greatest decrease in epithelial loss was recorded in **G4-D12-Asp**. No statistically significant difference was detected between **G2-D6-Asp** and **G3-D9-Asp**. In comparison to the other groups, the aspirin group showed the highest score of hemorrhage (Figure 20). All other groups showed significant decrease in the score of hemorrhage. **G3-D9-Asp** and **G4-D12-Asp** showed significant reduction in hemorrhage in comparison to the aspirin group. No significant difference was detected between **G1-D3-Asp** and **G2-D3-Asp** or between **G2-D6-Asp** and **G3-D9-Asp**.

**Figure 21:**
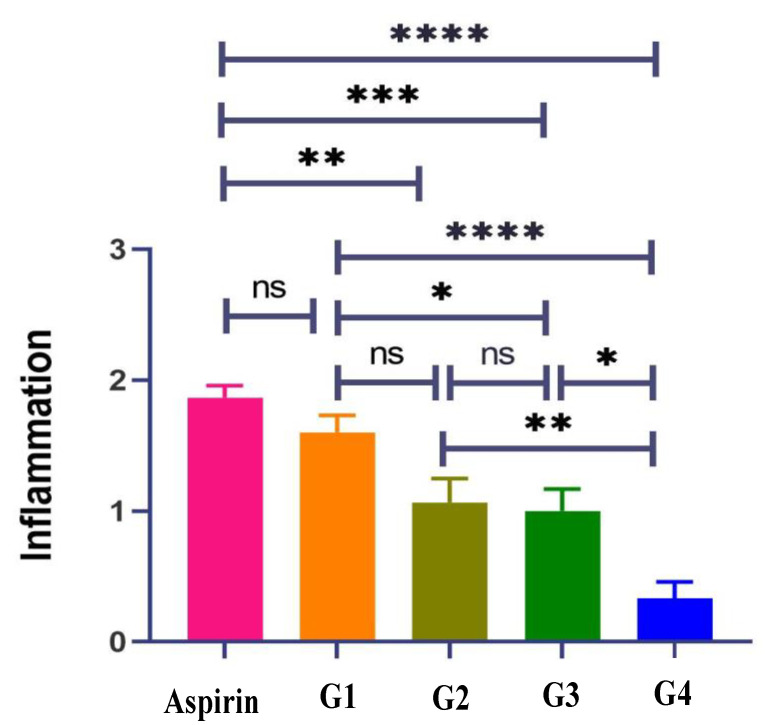
Inflammation score. Data were presented as means ± SE. * statistically significant at *p* ≤ 0.1, ** *p* *≤* 0.01, *** *p* ≤ 0.001, **** *p* ≤ 0.0001. Significance was considered at *p* ˂ 0.05. ns-not significant.

**Figure 22:**
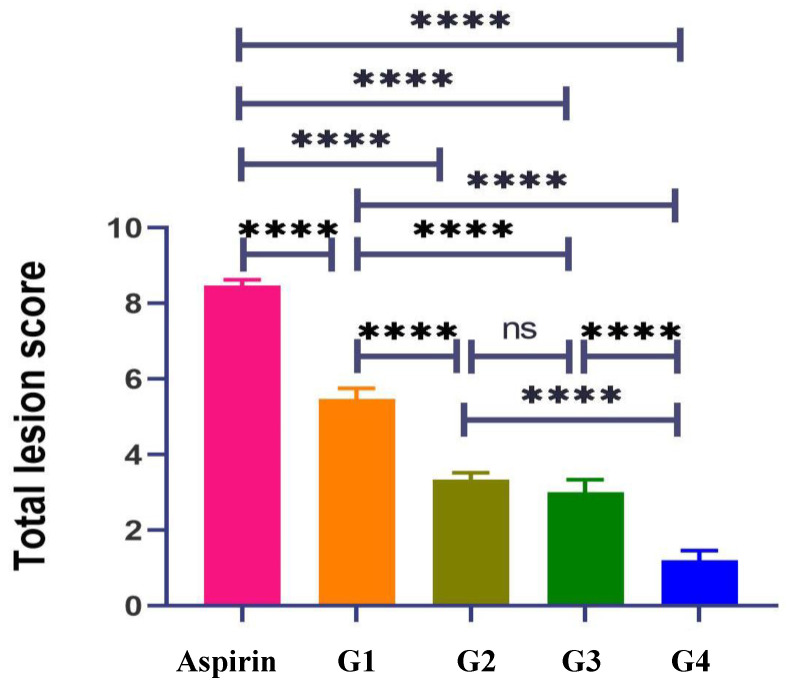
Total histologic score. Data were presented as means ± SE. **** *p*≤ 0.0001. Significance was considered at *p* ˂ 0.05. ns-not significant.

The inflammation score (Figure 21) also increased significantly in the aspirin group. No significant difference was observed between aspirin and **G1-D3-Asp**. **G2-D6-Asp**, **G3-D9-Asp,** and **G4-D12-Asp** showed significant reduction in the inflammation score. The least significant decrease was detected in **G4-D12-Asp**. Generally, the total histologic score (Figure 22) was increased significantly in the aspirin group in comparison to the other groups. All aspirin-based dendrimer complexes showed significant reduction in the histologic score in comparison to the aspirin group. **G4-D12-Asp** scored the least value in comparison to the other groups. No statistically significant difference was recorded between **G2-D6-Asp** and **G3-D9-Asp**.

## 5. Conclusions

A new oral drug delivery system based on organometallic dendrimers was successfully synthesized. Biologically active molecules were exploited to synthesize the aspirin-based dendrimer system containing 90 iron atoms. The antimicrobial activity against most types of microorganisms was increased for the four complexes by increasing the dendrimer generation. The in vitro and in vivo anti-inflammatory assessment of the aspirin-derived dendrimers was assessed using COX-1 and COX-2 ovine kit and carrageenan-induced inflammation in rats. It was noticed that lower generations of dendrimers showed less IC_50_ of COX-2 and hence better anti-inflammatory effect while keeping a similar selectivity index to aspirin. All aspirin-based dendrimers reduced paw edema and PGE-2 level; however, the second generation **G2-D6-Asp** showed the most potent anti-inflammatory effect among the tested generations. Results of gastrointestinal toxicity revealed that the ulcer index was significantly reduced after coordination of dendrimers with aspirin, with **G1-D3-Asp** showing the least ulcer index followed by **G4-D12-Asp**. The histopathology examination of dissected stomach showed that increasing the generation of dendrimers reduced the toxicity on stomach tissue, as evidenced by reduced inflammation, hemorrhage, and epithelial loss. It is thus possible to conclude that the second generation **G2-D6-Asp** showed the best efficacy and least toxicity compared to other generations as it showed similar anti-inflammatory effect to aspirin but with less toxicity on the stomach.

## Figures and Tables

**Table 1:** The results of antimicrobial activity for the aspirin and **G1-D3-Asp**, **G2-D6-Asp**, **G3-D9-Asp,** and **G4-D12-Asp** at 15 mg/mL against different strains of bacteria and fungi compared to references.

	G1-D3-Asp	G2-D6-Asp	G3-D9-Asp	G4-D12-Asp	Asp	Gentamicin	Ampicillin	Nystatin
*B. subtilis*	14 ± 0.11	16 ± 0.10	16 ± 0.09	20 ± 0.12	23 ± 0.08	-	25 ± 0.06	-
*S. aureus*	19 ± 0.09	21 ± 0.11	24 ± 0.10	28 ± 0.10	30 ± 0.07	-	15 ± 0.05	-
*E. coli*	10 ± 0.10	11 ± 0.09	13 ± 0.11	17 ± 0.11	18 ± 0.06	17 ± 0.04	-	-
*K. pneumoniae*	15 ± 0.08	19 ± 0.10	22 ± 0.10	23 ± 0.09	26 ± 0.11	22 ± 0.02	-	-
*C. albicans*	15 ± 0.09	19 ± 0.11	21 ± 0.07	21 ± 0.12	29 ± 0.09	-	-	21 ± 0.11
*A. niger*	18 ± 0.11	20 ± 0.09	21 ± 0.11	25 ± 0.11	30 ± 0.10	-	-	15 ± 0.08

All data are represented as mean ± SE.

**Table 2:** Cytotoxicity of **G1-G4** aspirin-based dendrimer and aspirin.

	G1-D3-Asp	G2-D6-Asp	G3-D9-Asp	G4-D12-Asp	Aspirin	Doxorubicin
Hep-G2	372.21 ± 0.09	324.95 ± 0.10	105.05 ± 0.05	111.77 ± 0.11	192.40 ± 0.04	4.80 ± 0.09
MCF-7	323.18 ± 0.07	320.59 ± 0.12	193.46 ± 0.04	191.66 ± 0.09	208.96 ± 0.05	5.70 ± 0.07
HEK-293	342.01 ± 0.06	320.48 ± 0.09	224.31 ± 0.07	234.11 ± 0.10	240.18 ± 0.03	205.07 ± 0.05

All data are represented as mean ± SE.

**Table 3:** IC_50_ of inhibition of COX-1/COX-2 enzyme of the tested compounds.

Code	COX-1 µm IC_50_	COX-2 µm IC_50_	SI *
Celecoxib	14.7	0.045	>300
Rofecoxib	14.5	0.025	>500
Indomethacin	0.1	0.080	>500
Diclofenac sodium	3.8	0.84	>4
Aspirin	1.3	5.9	0.22
G1-D3-Asp	1.5	6.8	0.22
G2-D6-Asp	1.3	6.5	0.20
G3-D9-Asp	1.3	6.4	0.20
G4-D12-Asp	1.2	6.3	0.19

* Selectivity index (COX-1 IC_50_/ COX-2 IC_50_).

**Table 4:** The effect of different treatments on carrageenan-induced paw edema at different intervals.

Groups	Paw Thickness (mm)				% Inhibition			
	1 h	2 h	4 h	6 h	1 h	2 h	4 h	6 h
Normal	3.11 ^b^ ± 0.10	3.19 ^b^ ± 0.16	3.19 ^b^ ± 0.22	3.14 ^b^ ± 0.20	-	-	-	-
Control	5.10 ± 0.20	6.92 ± 0.37	7.64 ± 0.42	9.65 ± 1.8	-	-	-	-
Aspirin	3.32 ^b^ ± 0.23	4.00 ^b^ ± 0.36	4.96 ^b^ ± 0.48	5.08 ^b^ ± 0.56	34.82	42.23	35.08	47.38
G1-D3-Asp	4.54 ^b^ ± 0.34	5.23 ^b^ ± 0.59	6.44 ^b^ ± 0.44	7.50 ^b^ ± 0.76	10.91	24.37	15.78	22.31
G2-D6-Asp	3.45 ^b^ ± 0.34	3.99 ^b^ ± 0.79	4.32 ^b^ ± 0.59	4.97 ^b^ ± 0.80	32.29	42.34	43.53	48.51
G3-D9-Asp	4.27 ^b^ ± 0.30	4.71 ^b^ ± 0.32	5.58 ^b^ ± 0.55	5.93 ^b^ ± 0.38	16.15	31.95	26.94	38.60
G4-D12-Asp	3.96 ^b^ ± 0.23	4.84 ^b^ ± 0.40	6.21 ^b^ ± 0.51	7.00 ^b^ ± 0.73	22.20	30.01	18.76	27.52

All data of the paw thickness are represented as mean ± SD. Statistical significance was measured at *p* < 0.05 based on one-way analysis of variance ANOVA test followed by Tukey’s test for multiple comparisons. ^**b**^ was significantly different from the control group at *p* < 0.05.

**Table 5:** In vivo study of rat serum PGE2.

Compound No.	PGE2	
	Mean Serum Concentration (Pg/mL)	Inhibition%
Normal	122.72 ^b^ ± 5.53	-
Control	293.68 ± 13.78	-
Aspirin	81.051 ^b^ ± 14.39	72.40
G1-D3-Asp	190.22 ^b^ ± 20.44	35.23
G2-D6-Asp	103.30 ^b^ ± 22.76	64.83
G3-D9-Asp	139.51 ^b^ ± 18.82	52.50
G4-D12-Asp	184.05 ^b^ ± 29.67	37.33

All data of the PGE2 concentration are represented as mean ± SD. Statistical significance was measured at *p* < 0.05 based on one-way analysis of variance ANOVA test followed by Tukey’s test for multiple comparisons. ^**b**^ was significantly different from the control group at *p* < 0.05.

**Table 6:** Evaluation of the gastric mucosal lesions of the tested Aspirin-based dendrimers.

Compound No.	Gastric Mucosal Lesions		
	Ulcer Area (mm)	Ulcer Score	Ulcer Index
Normal	0.00 ± 0.00	0.00 ± 0.00	0.00 ± 0.00
Aspirin	86.91 ± 9.51	5.00 ± 0.00	1.45 ^a^ ± 0.16
G1-D3-Asp	5.17 ± 1.13	1.17 ± 0.41	0.09 ^b^ ± 0.02
G2-D6-Asp	16.78 ± 2.71	3.17 ± 0.41	0.28 ^a,b^ ± 0.04
G3-D9-Asp	17.98 ± 0.93	3.00 ± 0.00	0.30 ^a,b^ ± 0.02
G4-D12-Asp	7.58 ± 1.00	2.00 ± 0.00	0.13 ^a,b^ ± 0.02

All data are represented as mean ± standard deviation. Statistical significance was considered at *p* < 0.05 based on one-way analysis of variance ANOVA test followed by Tukey’s test for multiple comparisons. ^**a**^ significantly different from normal at *p* < 0.05. ^**b**^ was significantly different from aspirin group at *p* < 0.05.
